# Heat-induced SIRT1-mediated H4K16ac deacetylation impairs resection and SMARCAD1 recruitment to double strand breaks

**DOI:** 10.1016/j.isci.2022.104142

**Published:** 2022-03-23

**Authors:** Sharmistha Chakraborty, Mayank Singh, Raj K. Pandita, Vipin Singh, Calvin S.C. Lo, Fransisca Leonard, Nobuo Horikoshi, Eduardo G. Moros, Deblina Guha, Clayton R. Hunt, Eric Chau, Kazi M. Ahmed, Prayas Sethi, Vijaya Charaka, Biana Godin, Kalpana Makhijani, Harry Scherthan, Jeanette Deck, Michael Hausmann, Arjamand Mushtaq, Mohammad Altaf, Kenneth S. Ramos, Krishna M. Bhat, Nitika Taneja, Chandrima Das, Tej K. Pandita

**Affiliations:** 1 Department of Radiation Oncology, Houston Methodist Research Institute, Houston, TX 77030, USA; 2 Department of Radiation Oncology, University of Texas Southwestern Medical Centre, Dallas, TX, USA; 3 Department of Medical Oncology, All India Institute of Medical Sciences, New Delhi 110029, India; 4 Department of Molecular and Cellular Biology, Baylor College of Medicine, Houston, TX 77030, USA; 5 Departments of Radiation Oncology, Washington University, St Louis, MO, USA; 6 Biophysics & Structural Genomics Division Saha Institute of Nuclear Physics, Bidhan Nagar, Kolkata, West Bengal 700064, India; 7 Homi Bhaba National Institute, Mumbai, India; 8 Department of Molecular Genetics, Erasmus MC Cancer Institute, Erasmus University Medical Center, 3000 Rotterdam, CA, the Netherlands; 9 Departments of Radiation Oncology, Moffitt Cancer Center, Tampa, FL 33612, USA; 10 Department of Molecular Medicine, University of South Florida, Tampa, FL 33612, USA; 11 Bundeswehr Institute of Radiobiology Affiliated to the University of Ulm, Neuherbergstr. 11, 80937 Munich, Germany; 12 Kirchhoff-Institute for Physics, Heidelberg University, Im Neuenheimer Feld 227, 69120 Heidelberg, Germany; 13 Department of Biotechnology, University of Kashmir, Srinagar, Jammu and Kashmir 190006, India; 14 Center for Genomics and Precision Medicine, Texas A&M College of Medicine, Houston, TX, USA

**Keywords:** Biological sciences, Molecular biology, Epigenetics

## Abstract

Hyperthermia inhibits DNA double-strand break (DSB) repair that utilizes homologous recombination (HR) pathway by a poorly defined mechanism(s); however, the mechanisms for this inhibition remain unclear. Here we report that hyperthermia decreases H4K16 acetylation (H4K16ac), an epigenetic modification essential for genome stability and transcription. Heat-induced reduction in H4K16ac was detected in humans, *Drosophila*, and yeast, indicating that this is a highly conserved response. The examination of histone deacetylase recruitment to chromatin after heat-shock identified SIRT1 as the major deacetylase subsequently enriched at gene-rich regions. Heat-induced SIRT1 recruitment was antagonized by chromatin remodeler SMARCAD1 depletion and, like hyperthermia, the depletion of the SMARCAD1 or combination of the two impaired DNA end resection and increased replication stress. Altered repair protein recruitment was associated with heat-shock-induced γ-H2AX chromatin changes and DSB repair processing. These results support a novel mechanism whereby hyperthermia impacts chromatin organization owing to H4K16ac deacetylation, negatively affecting the HR-dependent DSB repair.

## Highlights

• H4K16ac deacetylation during hyperthermia is conserved in human, *Drosophila*, and yeast
• Dynamic regulation of the chromatin functions during hyperthermia is SIRT1-dependent
• SIRT1 function is negatively impacted by SMARCAD1
• Hyperthermia increases replication stress and impacts DNA resection, impairing DSB repair

## Graphical abstract

**Figure:**
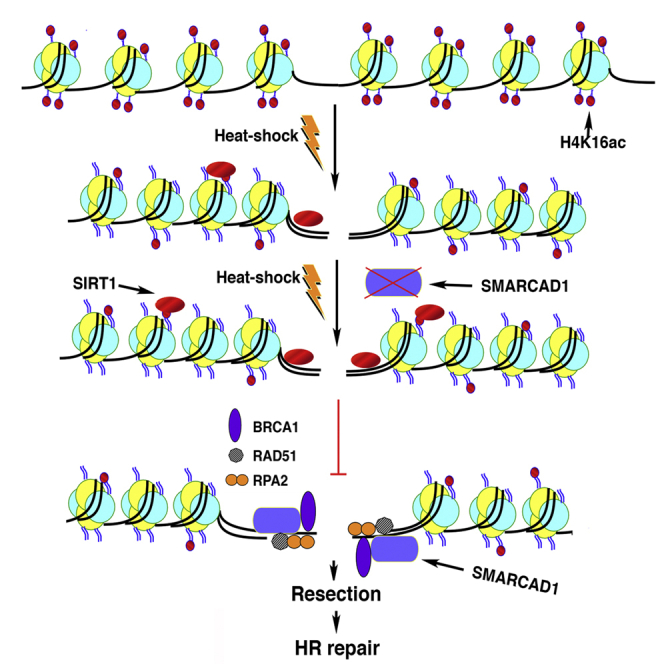

## Introduction

Exposure to elevated temperatures can cause heat stroke, neurological, and cardiovascular diseases, as well as induce deaths from heart attacks (www.epa.gov/climate-indicators; Li et al., 2020). It has been estimated that about 166,000 people died from heat-stress worldwide during 1998–2017 (www.who.int) (Witze, 2021). In the United States alone, 10,649 residents died from weather-related conditions, with 34% of these deaths attributed to hyperthermia during 2006–2010 (Murphy et al., 2013). In recent decades, global warming has led to a rise in summer temperatures, and extreme periods of elevated heat are expected to become more persistent and frequent (Kumar and Singh, 2021). Adults aged ≥65 years are at a higher-than-average risk of heat-related death (Zanobetti et al., 2012) and form a rapidly increasing proportion of the population. Moreover, children are also particularly vulnerable to heat-related illness and death (Hajat et al., 2005).

Histone post-translational modifications are particularly labile and therefore provide a mechanism for organisms to rapidly respond to acute environmental stresses (Maunakea et al., 2010). One of the most common histone modifications is H4K16 acetylation (H4K16ac), which marks active genes and enhancers in embryonic stem cells (Gupta et al., 2008; Horikoshi et al., 2013; Taylor et al., 2013). This modification constrains the formation of higher order chromatin structures, possibly by forcing a more open chromatin configuration that facilitates the access of various proteins to DNA (Shogren-Knaak et al., 2006). Such chromatin remodeling is a critical requirement for most DNA-dependent processes such as the assembly of transcription or DNA damage response (DDR) components (Hunt et al., 2013; Singh et al., 2020). H4K16ac is differentially regulated by the histone acetyltransferase, MOF (Akhtar and Becker, 2000; Gupta et al., 2005; Smith et al., 2005; Taipale et al., 2005), and the corresponding deacetylases, SIRT1/2 (Rifai et al., 2018; Vaquero et al., 2006). These enzymes play a crucial role in multiple biological processes, including cellular development and differentiation (Li et al., 2012), maintenance of metabolic homeostasis (Ryall et al., 2015), neuronal function (Dmitriev and Papkovsky, 2015) and lifespan regulation (Dang et al., 2009). Altered function of these proteins leads to developmental defects (Gupta et al., 2008; Taylor et al., 2013), metabolic disorders (Hussein et al., 2020), neurological disorders (An et al., 2020; Chen et al., 2020; Sheikh et al., 2020), and aging defects (Dang et al., 2009). Here, we report that heat-induced deacetylation of H4K16ac is SIRT1-dependent and enhanced by depletion of SMARCAD1, a member of the SNF2-family of chromatin-remodelers, resulting in replication stress and impaired repair of DNA damage by HR.

## Results

### Hyperthermia reduces H4K16ac levels

**Figure 1:**
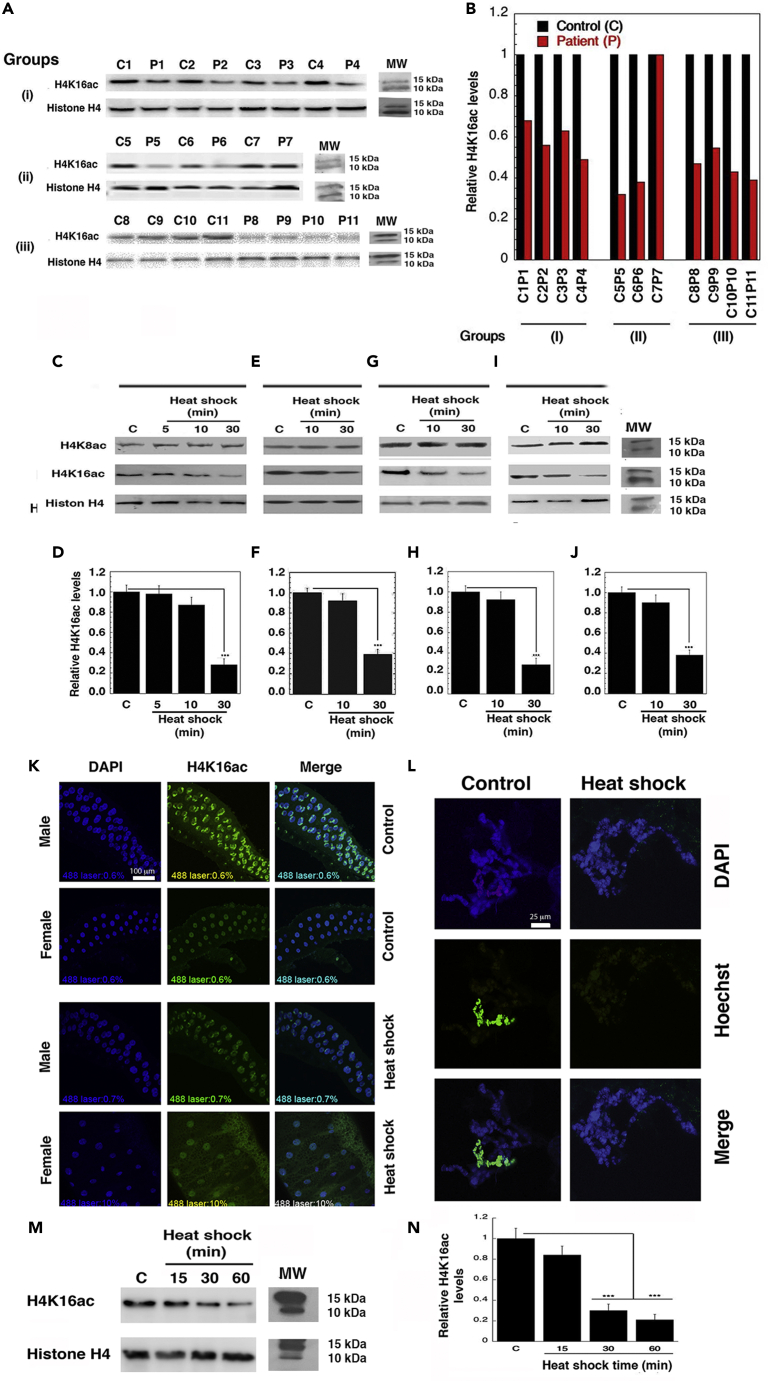
Hyperthermia and heat-shock reduce cellular H4K16ac levels (A and B) Hyperthermia patients have significant reduction in H4K16ac levels in eleven hyperthermia patient cohort (P1–P11) and matched control cohort (C1–C11) were analyzed (A) and quantified (B). (C–F) Human cell lines H1299 (C and D) and HCT116 (E and F) were subjected to heat treatment of 43°C for 5, 10, and 30 min. There is no significant change in H4K8ac and a significant reduction in H4K16ac in both the cell lines (C–F) (∗∗∗*p* < 0.001). (G–J) Human xenotransplants with a small animal ultrasound (G and H) or water bath limb immersion device (I and J) showed a similar reduction in H4K16ac (∗∗∗*p* < 0.001). (K) Immunostaining analysis from *Drosophila* male and female whole third instar larval salivary gland with and without heat-shock. The absolute H4K16ac levels in male *Drosophila* X chromosome were higher than in females; hence, the laser power for capturing the H4K16ac levels in females post-heat-shock had to be increased to 10% (scale bar is 100 μm). (L) H4K16ac immune-staining of male *Drosophila* polytene salivary gland X chromosome of control and heat-shock treated (scale bar is 25 μm). (M and N) Western blot showing effect of heat-shock for 15, 30, and 60 min on H4K16ac levels in *Schizosaccharomyces pombe* analyzed (M) and quantified (N).

Lysine 16 is an essential acetylation site on the histone H4 N-terminal tail that regulates chromatin structure compaction (Shogren-Knaak et al., 2006). Hypoacetylation of K16 delays recruitment of repair-related proteins to DNA damage sites (Horikoshi et al., 2019; Gupta et al., 2008; Krishnan et al., 2011; Sharma et al., 2010). To determine the impact of elevated temperature on histone H4K16ac levels in humans, we analyzed peripheral blood mononuclear cells (PBMCs) from patients undergoing hyperthermia treatment (∼40.5°C) as compared with age- and sex-matched control individuals. About 90% of hyperthermia patients had a significant decrease in H4K16ac without any change in histone H4 levels (Figures 1A and 1B). Cultured cell lines (HeLa, HCT116, HEK293, H1299, U2OS, CHO) also manifested a similar significant reduction in H4K16ac levels, but not in H4K8ac levels (at 41–45°C and 43°C for 30 min) (Figures 1C–1F and S1A–S1C). Immunostaining for H4K16ac in HeLa cells also confirmed these Western blot findings (Figure S1D). In a mouse human xenotransplant model, heating with a small animal ultrasound or water bath limb immersion device *in vivo* was also found to reduce H4K16ac levels (Locke et al., 2005) (Figures 1G–1J). Finally, in organisms as diverse as *Drosophila* (Figures 1J, 1K, and S1E), and yeast (Figures 1M, 1N, S1F, and S1G), heat-shock also reduced H4K16ac levels, further indicating that the loss of H4K16ac is a general response to elevated temperature.

Depletion of MOF has been shown to reduce H4K16ac levels and enhance cell killing by ionizing radiation (IR) or DNA crosslinking agents (Gupta et al., 2005, 2008; Singh et al., 2018). Heat-shock increased IR-induced cell killing (Figures S2A and S2B) and in combination with MOF depletion (Figure S2C) further enhanced IR-induced cell killing (Figure S2D). Whereas heat-shock alone failed to increase chromosome aberrations, the latter were elevated by IR-exposure (Figure S2E).

**Figure 2:**
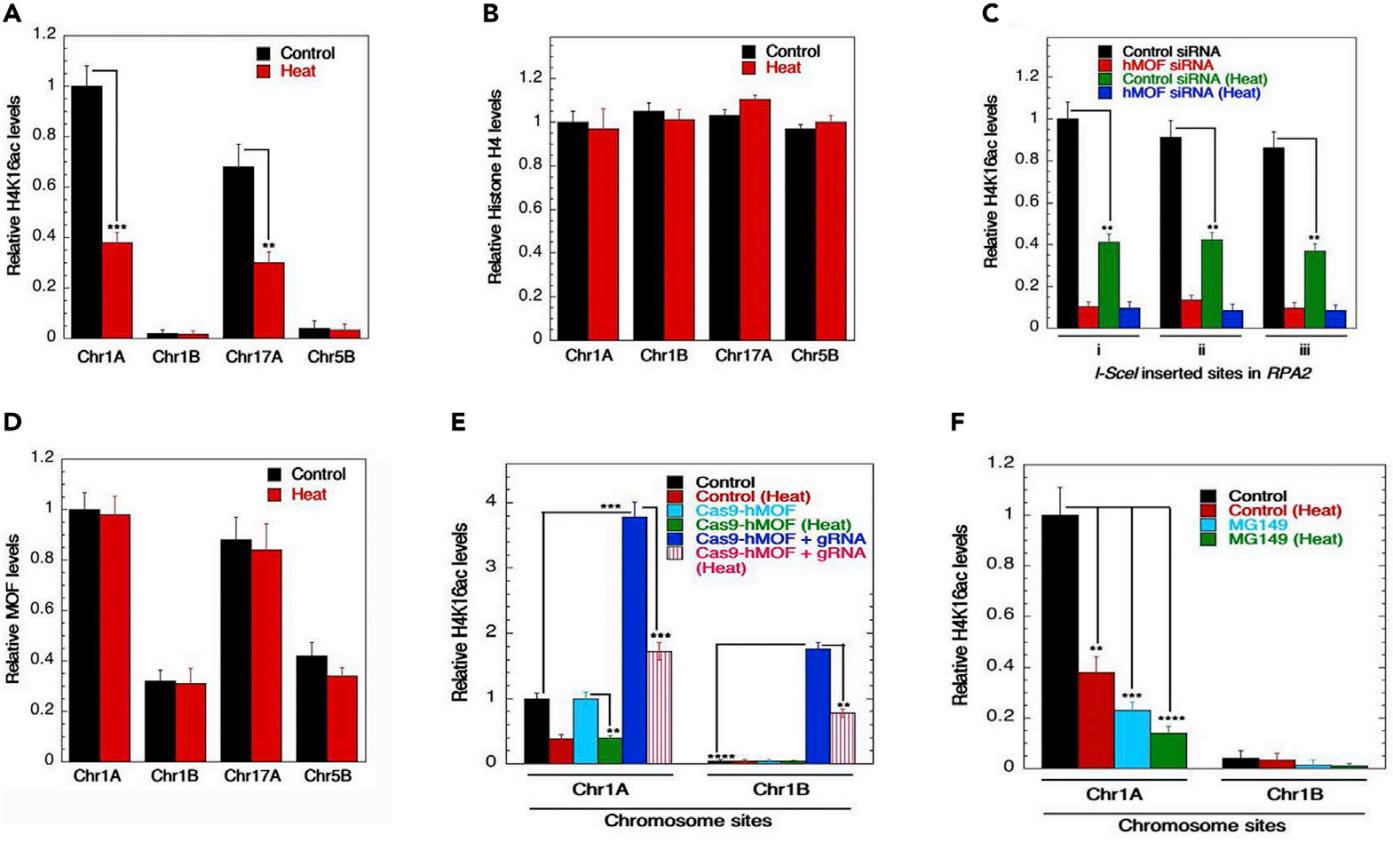
Effect of heat-shock on H4K16ac and MOF levels in gene-rich and gene-poor regions as determined by ChIP assay (A) Hyperthermia reduces H4K16ac levels in gene-rich (Chr1A, Chr17A) regions. (B) ChIP assays show no significant change in occupancy of H4 in gene-rich (Chr1A, Chr17A) or gene-poor (Chr1B, Chr5B) regions. (C) H4K16Ac occupancy was quantified in three independent regions of i-Sce1 inserted sites in RPA2 gene in the presence and absence of hMOF upon hyperthermia induction. Whereas heat or hMOF depletion alone led to a reduction in H4K16Ac occupancy, no further changes in H4K16Ac occupancy could be observed when combining the two. (D) The occupancy of hMOF showed no significant alteration in gene-rich (Chr1A, Chr17A) or gene-poor (Chr1B, Chr5B) regions. (E and F) H4K16ac occupancy was examined in Chr1A (gene-rich region) and Chr1B (gene-poor region) upon Cas9-mediated recruitment of hMOF upon heat treatment (E). Similarly, H4K16ac levels at Chr1A upon treatment with MOF inhibitor MG149 alone or in combination with heat treatment was examined (F) (∗*p* < 0.05; ∗∗*p* < 0.01; ∗∗∗*p* < 0.001; ∗∗∗∗*p* < 0.0001, determined by the chi-square test).

We previously mapped the genome-wide distribution of H4K16ac sites and their relationship with gene expression in human cells (Horikoshi et al., 2013). Pre-existing H4K16ac levels in gene-rich regions impact DNA DSB repair by the homologous recombination (HR) pathway, whereas hyperthermia also impairs DSB repair by HR (Horikoshi et al., 2019; Krawczyk et al., 2011). We examined whether heat-shock is associated with deacetylation in gene-rich regions by examining four specific sites located on three different chromosomes (namely Chr1A, Chr1B, Chr17A, and Chr5B) in engineered human cell lines as described previously (Horikoshi et al., 2019). Sites Chr1A and Chr17A are gene/H4K16ac-rich, whereas Chr1B and Chr5B are gene/H4K16ac-poor (Horikoshi et al., 2013, 2019). Hyperthermia reduced H4K16ac levels at gene/H4K16ac-rich regions as determined by ChIP (Figure 2A), without altering the overall histone H4 levels (Figure 2B). Furthermore, we determined the effect of heat-shock on H4K16ac levels within the *RPA2* gene, a constitutively expressed gene, in H1299 cells at three different defined sites described previously (Horikoshi et al., 2019). We observed that heat-shock reduced H4K16ac levels at all three gene sites (Figure 2C). Acetylation of H4K16 is primarily carried out by MOF (Gupta et al., 2005; Taipale et al., 2005), but heat-shock had a negligible effect on its association with the chromatin DSB sites as measured by ChIP (Figure 2D).

To determine whether the heat-induced reduction in H4K16ac levels can occur at chromosome sites where H4K16ac levels have been synthetically increased, we expressed Cas9-hMOF targeted to gene-rich (Chr1A) as well as gene-poor (Chr1B) regions (Figure 2E). This approach increased basal H4K16ac levels at either site and both were then reduced upon hyperthermia. Consistent with these results, the treatment of heat-shocked cells with the MOF inhibitor (MG149) (Valerio et al., 2017; van den Bosch et al., 2017) further decreased H4K16ac levels in the gene-rich region as compared with cells either treated with MG149 or heat alone (Figure 2F).

### Heat-shock-induced enrichment of SIRT1 at H4K16ac/gene-rich regions

**Figure 3:**
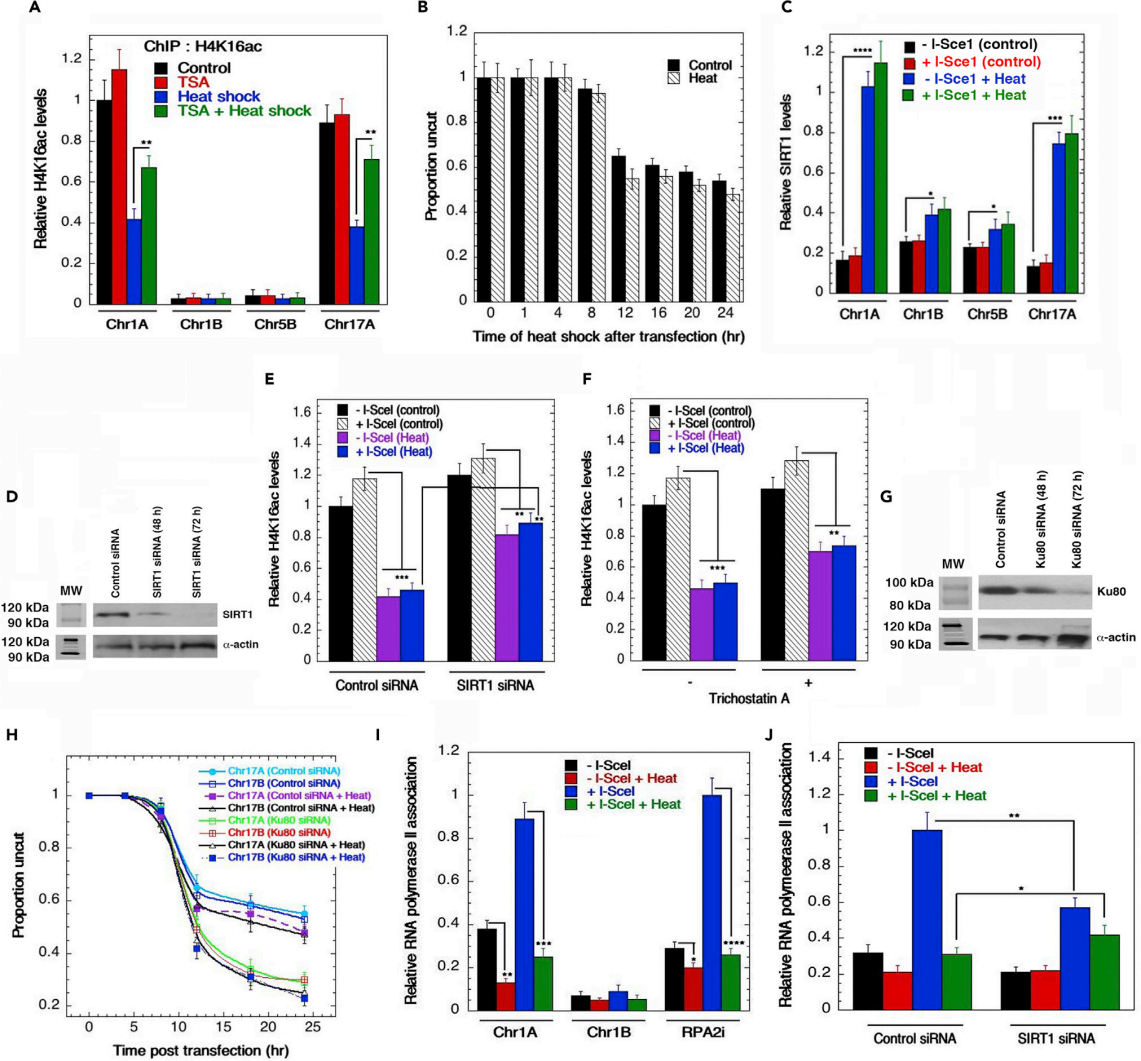
Impact of heat-shock on SIRTI-mediated H4K16ac deacetylation and DSB induction (A) Treatment with the deacetylase inhibitor trichostatin A (TSA) partially rescued the heat-shock-induced deacetylation of H4K16ac. (B) Heat-shock does not affect I-Scel-induced DNA DSBs. (C) Heat-shock leads to increased recruitment of SIRT1 in gene rich-regions (Chr1A and Chr17A). A combination of heat-shock and I-Scel-induced DNA DSBs does not further enrich H4K16Ac levels. (D) Western blot showing siRNA-mediated knockdown of SIRT1. (E) Compared with the control set with I-Scel-induced DNA DSBs, heat-shock leads to a reduction in the H4K16ac level. (F) Employing Trichostatin (TSA) as an HDAC inhibitor, the reduction in H4K16ac levels upon heat-shock treatment indicates that there is involvement of deacetylases in this process. (G) Western blot showing knockdown of Ku80. (H)The proportion of I-SceI-induced DNA DSBs with and without heat exposure was similar statistically. Knocking down Ku80 led to a reduction in the proportion of uncut DNA, although further reduction upon heat stress induction was not observed. (I and J) Association of RNA Polymerase II to the gene-rich regions was augmented upon I-Scel induced DNA DSBs (I). Invoking heat stress significantly reduces RNA Polymerase II association with the DSB site (I). SIRT1 knockdown leads to a reduction in RNA Polymerase association with the DSB site (I). The heat-shock induction in absence of SIRT1 could partially rescue RNA Polymerase ll occupancy (I) (∗*p* < 0.05; ∗∗*p* < 0.01; ∗∗∗*p* < 0.001; ∗∗∗∗*p* < 0.0001, determined by the chi-square test).

The deacetylase inhibitor Trichostatin A (TSA) treatment has been reported to increase H4K16ac levels (Dmitriev and Papkovsky, 2015). We noted that TSA treatment rescued H4K16ac from the heat-shock-induced deacetylation (Figure 3A). Interestingly, an interaction between SIRT proteins and H4K16ac has been observed in budding yeast heterochromatin DNA fibers (Swygert et al., 2018). In addition, hMOF and the SIRT1 sirtuin reportedly have opposite effects on H4K16ac levels (Hajji et al., 2010). In the cellular response to environmental stress, Sirtuins promote DNA repair as well as apoptosis by modulating post-translational modifications (Guarente, 1999; Rodriguez et al., 2013; Soni et al., 2021; Vaziri et al., 2001) with SIRT6 being required for efficient DSB repair in rodents (Tian et al., 2019). Whereas heat-shock was not found to change the levels of SIRT1, SIRT2, SIRT4, or SIRT6 (Figures S3A and S3B), SIRT1 was enriched at gene-rich regions (Chr1A, Chr17A) following heat-shock as determined by chromatin immunoprecipitation (ChIP) (Figures S3C–S3E). In contrast to SIRT1 (Figure S3C), heat-shock decreased SIRT2 and SIRT4 association with the gene-rich region (Chr1A), whereas SIRT6 association remained unchanged (Figures S3D–S3F). To determine the impact of heat-shock and DSBs on SIRT1 recruitment, we examined the effect of heat-shock on l-Sce1 (an endonuclease that recognizes and cleaves within an 18-base-pair target) induced DSBs and found that heat-shock had no impact on DNA cleavage (Figure 3B). SIRT1 levels, however, increased significantly at DSB sites in H4K16ac/gene-rich regions (Chr1A, Chr17A) upon heat-shock (Figure 3C). Furthermore, we observed that SIRT1 depletion (Figure 3D) partially rescued the heat-induced reduction in H4K16ac levels (Figure 3E), with similar results observed after TSA treatment (Figure 3F). Enrichment of SIRT1 did not impact DSB induction as the proportion of uncut DNA in the presence or absence of a non-homologous end-joining (NHEJ) DNA repair pathway protein Ku80 was unchanged (Figure 3G) nor was the relative amount of uncut DNA in gene-rich and gene-poor regions altered by heat-shock (Figure 3H). However, the significant increase in RNA Polymerase ll (RNAPll) recruitment after DSB induction was substantially decreased upon heat-shock in gene-rich region of Chr1A (as well as within the *RPA*2i gene) (Figure 3I). Depletion of SIRT1 impacted heat-induced deacetylation of H4K16ac (Figure 3E), which correlates with reduced RNAPolll recruitment at the DSB (Figure 3J). Furthermore, we found that heat-shock reduces RNAPll enrichment at DSB sites in SIRT1-depleted cells (Figure 3J).

### Heat-shock affects recruitment of DSB repair factors

**Figure 4:**
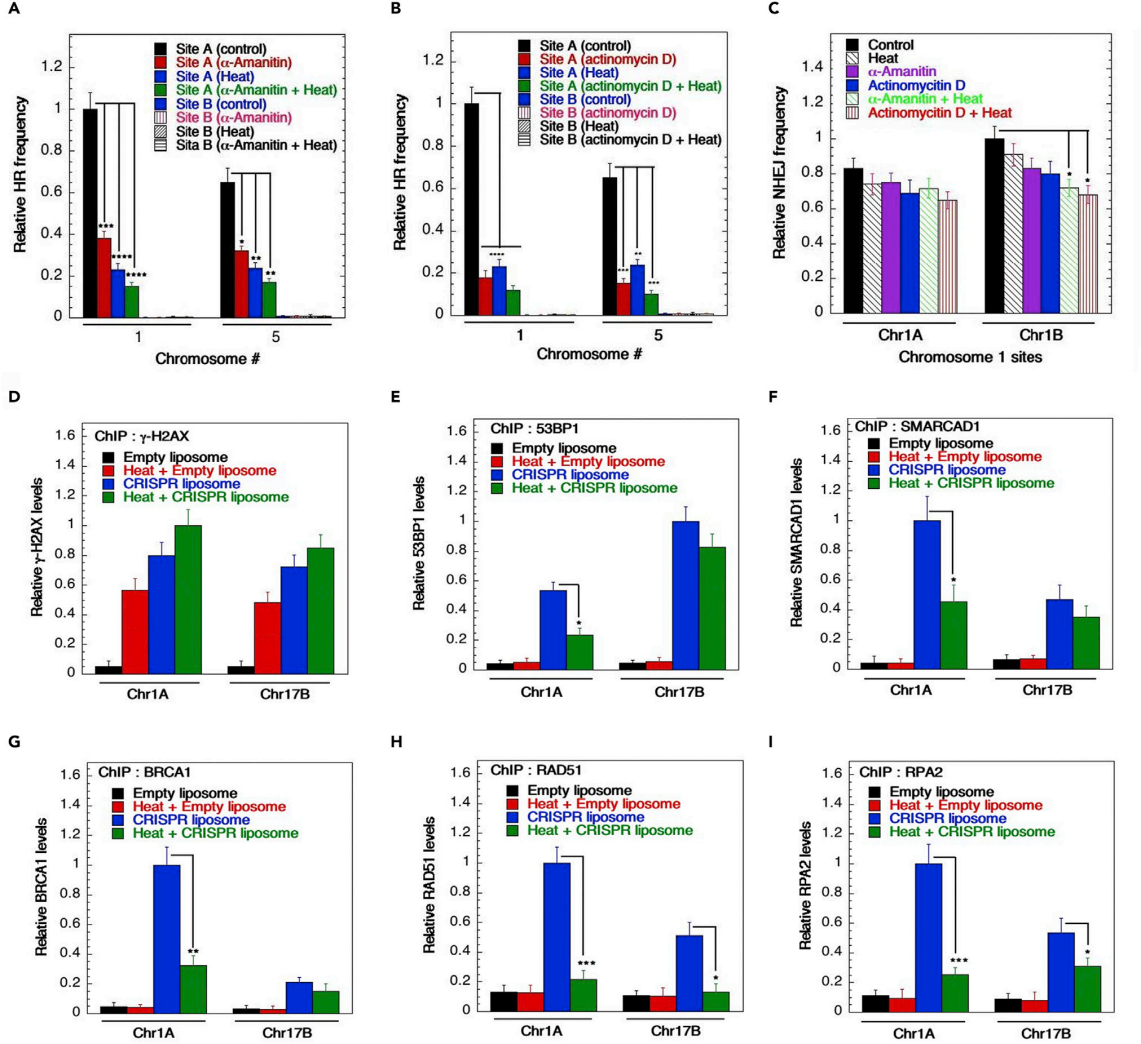
Heat-shock impacts recruitment of DSB repair factors in gene-rich regions (A and B) Transcription blockers [α-amanitin (A) or actinomycin D (B)] enhance the heat-shock effect on the HR frequency. A combination of the transcription blocker and heat stress leads to a significant reduction in the HR frequency determined at Chr1A, Chr1B, Chr5A, and Chr5B. (C) The NHEJ frequency was not significantly altered upon heat stress induction in combination with transcription blockers in both gene-rich and gene-poor regions. (D–I) The occupancy of the DDR factors γ-H2AX (D), 53BP1 (E), SAMRCAD1 (F), BRCA1 (G), RAD51 (H), and RPA2 (I) in gene-rich and gene-poor regions as a sequel to heat-shock and CRISPR liposome harboring gRNA to induce site-specific DSBs. Heat-shock led to enrichment of γ-H2AX, which further increased upon treatment with CRISPR liposome (D). In contrast, the heat-shock significantly reduced 53BP1 (E), SAMRCAD1 (F), and BRCA1 (G) occupancy at DSB sites most prominently in the gene-rich region. A reduction in RAD51 (H) and RPA2 (I) enrichment was seen at the DSB sites in both gene-rich and gene-poor regions (∗*p* < 0.05; ∗∗*p* < 0.01; ∗∗∗*p* < 0.001; ∗∗∗∗*p* < 0.0001, determined by the chi-square test).

We found that the relative frequency of DSB repair by HR was significantly reduced by heat-shock (Figure S4A). Transcriptional inhibitors are known to decrease HR and the combination of transcriptional inhibitors, actinomycin D, or α-amanitin with heat-shock further reduced HR (Figures 4A and 4B). Whereas actinomycin D or α-amanitin blocked transcription, and hyperthermia results in chromatin changes, there was no effect of heat or these inhibitors on NHEJ-mediated DSB repair (Figures 4C and S4B).

To determine the impact of heat on the recruitment of DSB repair-associated proteins, we measured and compared repair protein recruitment with DSBs in H4K16ac/gene-rich (Chr1A) and H4K16ac/gene-poor (Chr17B) regions before and after site-specific DSB induction by CRISPR-targeting. Treatment of cells with CRISPR liposomes significantly enriched for γ-H2AX, in gene-rich and gene-poor regions (Figure 4D). In contrast to heat-induced γ-H2AX enrichment at DSB sites, heat-shock significantly reduced 53BP1 occupancy at DSBs in gene-rich (Chr1A) as well as in gene-poor regions (Figure 4E). Similarly, heat-shock reduced SMARCAD1, BRCA1, RAD51, and RPA2 enrichment at DSB sites in the gene-rich and gene-poor regions (Figures 4F–4I). We confirmed that the reduced enrichment of DNA repair proteins at DSBs was not owing to heat-induced degradation of repair-associated proteins (such as Rad51, SMARCAD1, MOF) as determined by Western blot analysis (Figures S4C and S4D).

### Heat-shock impairs DNA replication fork progression and restart efficiency

**Figure 5:**
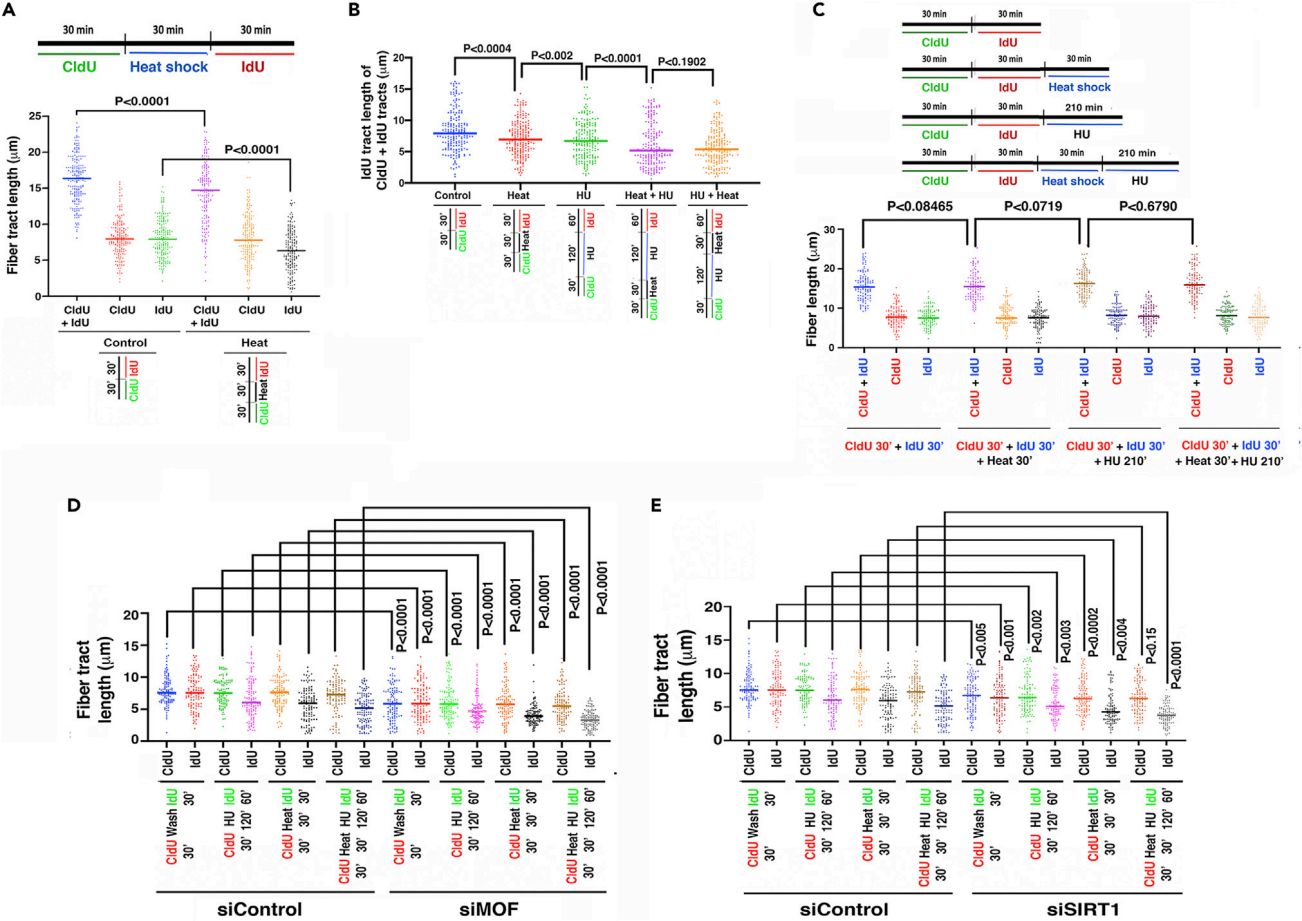
Heat-shock causes replication stress as measured by DNA fiber tract length (A) Heat-shock reduced fork progression significantly as determined by the reduction of second label fiber length. Fork progression was measured upon heat-shock before or after HU treatment (Scale bar is 10 μm). (B) The reduction in fork speed was more significant for an HU and heat stress combination as it is higher than each of them alone. (C) To examine the effect of heat or HU treatment on DNA degradation, cells were treated with heat or HU treatment in isolation or in combination following DNA synthesis. No reduction in the fiber length could be observed. (D and E) Fiber tract length measurements were done in MOF (D) or SIRT1 (E) silenced conditions. Upon MOF depletion, heat-shock in combination with HU treatment led to a reduced fiber tract length (D). Similar heat-shock and HU treatment combination also led to a reduced fiber tract length in the absence of SIRT1 (E).

Heat-shock induces replication stress and impairs genome integrity (Velichko et al., 2012); however, the mechanism(s) that regulate(s) these replication fork dynamics upon hyperthermia remains unclear. Heat-shock reduces H4K16 acetylation, whereas the depletion of the MOF causes DNA replication fork stalling (Singh et al., 2018); hence, we reasoned that heat-shock may cause replication fork stalling. We analyzed the impact of heat-shock on DNA replication fork progression using DNA fiber assays (Petermann et al., 2010; Singh et al., 2013, 2018) (Figure S5A). Consistent with a previous report (Velichko et al., 2012), we observed significant reductions in replication fork progression, enhanced fork stalling, and reduced firing of new origins upon heat-shock (Figures 5A and S5B–S5D). The heat-induced reduction in fork progression was further exacerbated when cells were already under replicative stress owing to treatment with hydroxyurea (HU), a drug that depletes the cellular nucleotide pool (Figure 5B). To investigate if the additive stress of heat can also lead to fork degradation, we treated cells after IdU labeling of newly synthesized DNA with heat or HU alone or in combination. No significant reduction in the IdU track could be observed, which would have been expected if the forks underwent degradation (Figures 5C and S5E). Together, these data suggest that heat stress impacts active, ongoing, and restarted replication fork stability (Lo et al., 2021) but not stalled fork protection (Schlacher et al., 2012).

### Impact of MOF and SIRT1 depletion on heat-induced replication stress

We have previously reported the involvement of MOF in suppression of replication stress (Singh et al., 2018) and, therefore, determined if MOF depletion and hyperthermia have a synergistic effect on replication stress. Heat-shock or MOF depletion alone significantly reduced the frequency of CldU + IdU co-labeled fibers, which are a measure of ongoing DNA replication (Figure S5F). MOF-depleted cells exposed to heat-shock showed increased replication stress compared with cells either depleted for MOF or treated with heat alone (Figure 5D). Although we observed that SIRT1 depletion partially rescued the heat-induced deacetylation of H4K16ac (Figure 3D), there was a significant increase in replication stress, which was further enhanced by heat-shock (Figure 5E). These data suggest that although the lack of SIRT1 prevents heat-induced H4K16ac deacetylation, SIRT1 is likely to play additional roles during replication stress.

### SMARCAD1 depletion is associated with heat-mediated enhancement of SIRT1 recruitment and replication stress

**Figure 6:**
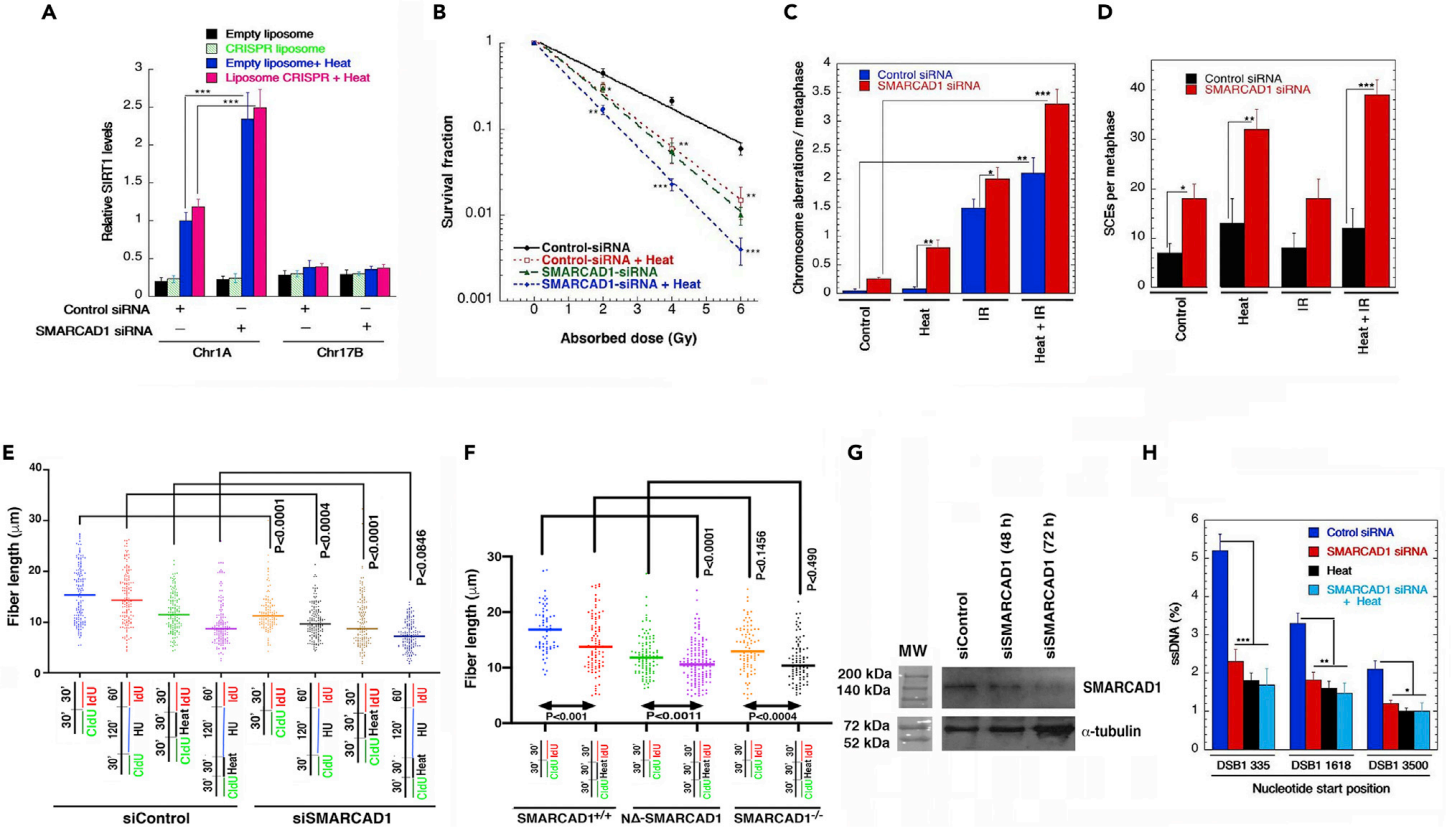
SMARCAD1 depletion enhances heat-mediated replication stress by enhancement of SIRT1 recruitment (A) SMARCAD1 depletion caused a significant heat induced increase in SIRT1 recruitment at the gene-rich site (Chr1A) as determined by ChIP. (B) SMARCAD1 depletion or heat-shock treatment led to reduced cell survival in clonogenic assays. A combination of the two further reduced the cell survival. (C and D) Increased chromosome aberrations are observed under SMARCAD1 depleted conditions, with a further increase upon heat-shock, IR treatment, or a combination of the two (C). Similar observations can also be noted during sister chromatid exchange (SCE). The effect of SMARCAD1 silencing alone and in combination with heat alone as well as heat and IR led to increased SCE during metaphase (D). (E and F) SMARCAD1 silencing alone or in a combination with heat and HU led to a reduction in the fiber length (E). SMARCAD1 depletion and heat-shock individually impacted the fiber track length (F). (G) Western blot showing knockdown of SMARCAD1 in U2OS cells by specific siRNA. (H) The effect of SMARCAD1 knockdown, heat, and a combination of the two on DNA end resection at DSBs (three regions distal from the actual break site) was measured. SMARCAD1 knockdown and heat impacted the DNA end resection but no further increase was observed after simultaneous treatment (∗*p* < 0.05; ∗∗p < 0.01; ∗∗∗p < 0.001, determined by the chi-square test).

SMARCAD1 chromatin remodeling factor is involved in the regulation of global histone acetylation during replication (Rowbotham et al., 2011), propagation and organization of epigenetic patterns after DNA replication (Rowbotham et al., 2011), as well as in heterochromatin maintenance and inheritance (Taneja et al., 2017). SMARCAD1 is an important component in HR DSB repair and is specifically involved in DNA end resection (Chakraborty et al., 2018). SMARCAD1 is recruited to DSBs and recruitment was impaired after heat-shock (Figure 4F). SMARCAD1-depletion also (Figure S6A) increases heat-mediated cell killing by IR as measured by clonogenic survival. We next examined the impact of SMARCAD1 depletion on heat-mediated recruitment of SIRT1 at the DSB sites in gene-rich/-poor sites. Heat-shock alone significantly enhanced SIRT1 recruitment at the DSB sites in the gene rich-region of Chr1A, as compared with the DSB site in the gene-poor region of Chr1B (Figure 6A). SIRT1 levels were markedly increased by SMARCAD1 depletion. No effect was observed in this assay when a different chromatin-binding SW1/SNF-related DNA repair protein, SMARCAL1, was depleted (Figures S6B and S6C).

A clonogenic assay showed reduced cell survival upon SMARCAD1 depletion or heat-shock and a further reduction upon a combination of the two (Figure 6B). Consistent with the reduced survival, an increased number of chromosome aberrations were observed in cells with depletion of SMARCAD1 along with heat-shock (Figures 6C and 6D). These cells also displayed significantly increased replication stress as compared with cells with either SMARCAD1 depletion or heat-shock alone (Figures S5A and S6E). Furthermore, SMARCAD1 depletion impacted fork stalling, with cells depleted for SMARCAD1 and exposed to heat having an increased frequency of stalled forks (Figure S6D), with no effect on the induction of new origins (Figure S6E).

A recent study using a separation-of-function mutant of SMARCAD1 (namely, NΔ-SMARCAD1), showed that it is efficient in HR repair but exhibits replication defects, reported a novel function of SMARCAD1 in maintaining active replication fork stability (Lo et al., 2021). Intriguingly, we also observed that heat-shock mainly impacts active replication fork stability rather than stalled fork protection (Figures S6D and S6E) and therefore addressed whether the heat-induced cell killing of SMARCAD1-depleted cells is only owing to DNA repair defects or also corresponding to replication fork stability defects. NΔ-SMARCAD1 (HR-proficient) as well as SMARCAD1-depleted (HR-deficient) cells exposed to heat-shock had a significant and similar defect in the fork progression rate (Figure 6F). The data above suggest that heat-shock affects the overall chromatin organization, further altering mechanisms of DNA repair and replication fork stability.

To examine in more detail heat-induced DSB repair affects, we measured DNA end resection at AsiSi-induced DSBs by quantifying ssDNA formation in U2OS cells stably expressing HA-ER-AsiSI (Chakraborty et al., 2018; Zhou et al., 2014) with and without heat-shock. DNA end resection efficiency was measured at three different distances from the actual break site. The first set of PCR primers spanned a region of 335 bp downstream of the break site to define short range resection, whereas the two other primer sets targeted for 1,618 and 3,500 bp downstream of the original break site defined the long-range DNA resection as measured by ssDNA formation. Depletion of SMARCAD1 (Figure 6G) and heat-shock individually impacted both short- and long-range resection with no further increases seen after simultaneous treatment (Figure 6H). This suggests that SMARCAD1 depletion and heat-shock are acting in the same resection pathway. The residual level of DSBs in such cells was associated with increased residual γ-H2AX, indicating that the depletion of SMARCAD1 and heat impair DNA DSB repair (Figures S6G and S6H), which agrees with stalled forks levels under these conditions.

### Effect of heat on repairosome foci formation

Whereas heat-shock causes deacetylation and impacts DNA repair, it is not known if heat impacts the compaction of chromatin to alter DNA repair. Studies in different model systems have indicated that chromatin undergoes significant dynamic changes in response to DNA damage, including local motion changes at damage sites and re-localization to new nuclear sites associated with specific repair pathways.

**Figure 7:**
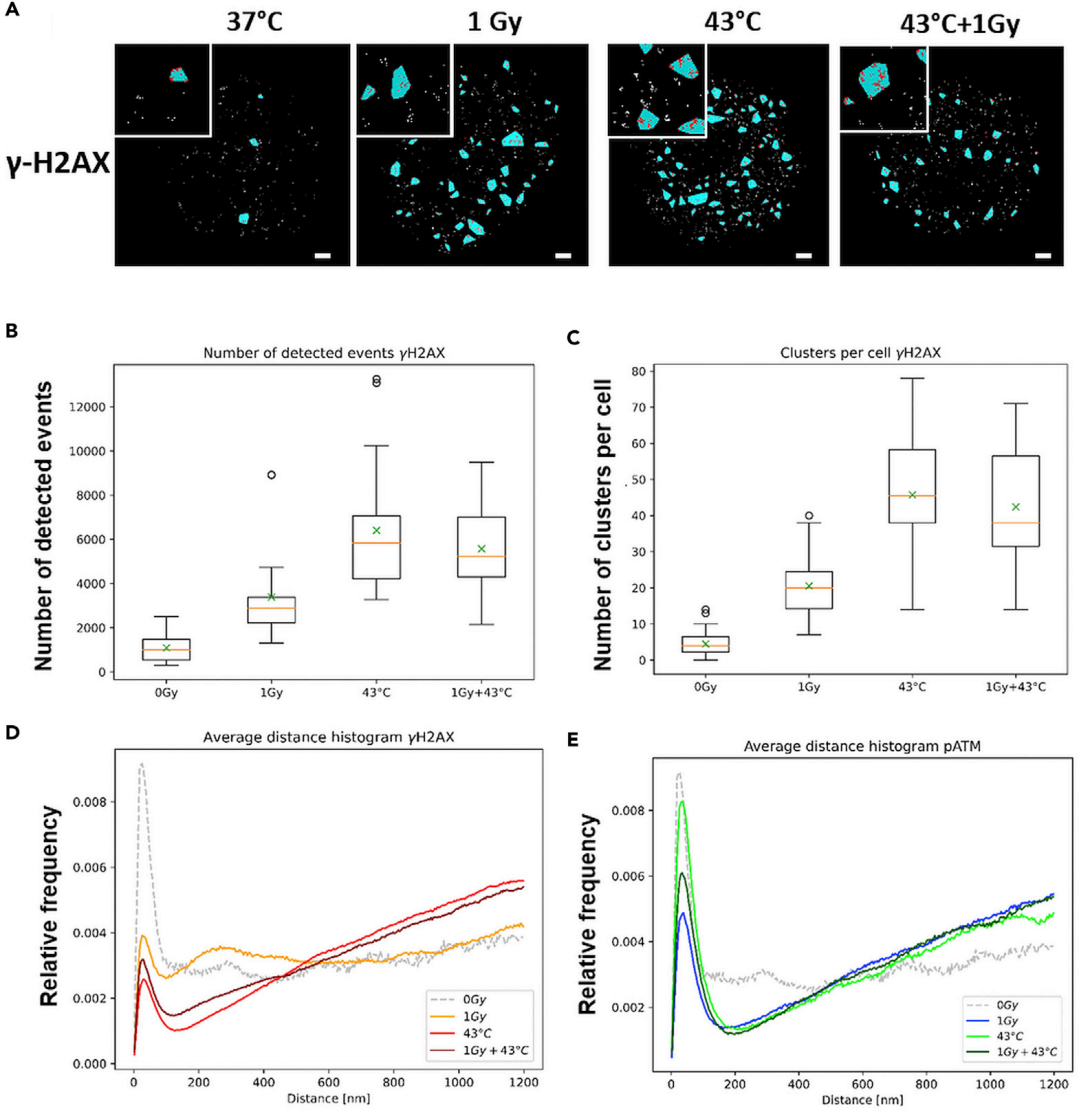
Heat-shock impairs repairosome foci cluster formation (A) Quantitative single molecule localization (SML) analysis of γ-H2AX signal tags in the chromatin of control, 1 Gy X-irradiated, and heat-shocked cells. Nuclear masks based on DAPI staining show the single molecule signal tags (red dots) and their clustering (light blue areas) in the differently treated nuclei (*n* = 20 for each condition) (scale bar is 1 μm). (B and C) Boxplots showing the average number of single molecule signal events for γ-H2AX per nucleus. (B) Normothermia exposed cells (0 Gy, 37°C) display the lowest number of events per nucleus, whereas 1Gy IR leads to a doubling of the signal numbers per nucleus. Hyperthermia induced an approximate six-fold increase in γ-H2AX SML signals per nucleus, whereas irradiation of heat-shocked cells did not further increase the number of γ-H2AX signals. (C) Analysis of clustering of γ-H2AX signals with the cluster parameters *R*_cluster_ = ≤ 200nm and *N*_min_ = 30 reveals that clusters are infrequent in non-exposed control cells, with 1 Gy IR inducing a three-fold increase in cluster numbers. HT alone induces a large number of clusters (∼45 per nucleus), which is not further increased by irradiation with 1 Gy. The boxes comprise the second and third quartile of the data. The mean values are marked by the “x” and the median by the orange line. The whiskers show the minimum or maximum of the dataset, with outliers (>± 2x SD) being marked as dots. (D and E) Nanoscale chromatin organization. Mean normalized frequency distributions according to Ripley’s *K* statistics of γ-H2AX (orange/red) and *p*-ATM (green, blue; see Figure S7 for frequency data) signal point distribution in nuclear chromatin. Tag distribution in control cells is stippled in grey. (D) γ-H2AX: spacing of nano-signals peaks around 20 nm, whereas 1 Gy-irradiated cells show increased distribution frequency with a “hump” at about 200–400 nm that likely reflects the clustering of γ-H2AX signals during microscopic focus formation. This is also seen for the occasional γ-H2AX foci of 0 Gy sham-irradiated cells. Heat-treatment abrogates the higher order clustering by a genome-wide random induction of γ-H2AX tags. (E) pATM: Mean normalized frequency distributions of the distances of pATM single molecule signals in HeLa cells show a peak around 20 nm and with clustering between 10 and 90 nm, thereafter increasing linearly, and suggesting a random distribution throughout nuclear chromatin.

To address the possibility that heat-shock leads to altered chromatin organization and non-DSB-related H2AX phosphorylation and altered chromatin organization, on and off DSBs, we next studied chromatin organization at nano-resolution by single molecule localization microscopy (SMLM; Scherthan et al., 2019; Zhang et al., 2015). The nanoscale distribution of γ-H2AX and active phospho-ATM (pATM) single molecule immunofluorescent signals was studied in the chromatin of heat-shock and/or irradiated HeLa cells (Figures 7A and S7A). Hyperthermia-induced more nano-γ-H2AX signals/nucleus relative to control (43°C vs. 37°C) (Figure 7B), which agrees with the previously reported global induction of γ-H2AX formation by heat-shock (Hunt et al., 2007). As expected, irradiated control cells (1 Gray) displayed an increase in clustered γ-H2AX signal tags (Figure 7C) (Hausmann et al., 2018). The average frequency of γ-H2AX signals/nucleus did not increase after X-irradiation of heat-shocked cells (Figure 7B), which was also the case for large γ-H2AX clusters (Figure 7C) and likely relates to the extensive phosphorylation of H2AX by heat-shock alone.

As heat-shock and irradiation activate ATM (Hunt et al., 2007), we also studied phospho-ATM nano distribution in chromatin (Figure S7A). Here, irradiation significantly increased pATM foci frequency as compared with non-irradiated controls relative to that of heat-shocked cells (Figures S7B and S7C). The latter suggests that DSBs induce accumulation of phospho-ATM at DSB sites even after heat-shock (Figures S7A and S7C).

The spatial nano-organization of γ-H2AX and pATM signal tags in the nucleus was further studied by Ripley’s distance-frequency analysis. At the nanoscale γ-H2AX signal distances, densities peaked around 20 nm (range 10–80 nm) under all conditions (Figure 7D), whereas irradiation exposure induced an increase of signals displaying spatial proximity of about 200–400 nm, which likely reflects the clustering of signals (formation of large foci) at DSB-surrounding chromatin (Figure 7D). This was also seen at the occasional DSB foci of sham-irradiated cells (Figure 7D). Heat treatment altered the nano and meso chromatin distribution such that the increased frequency of signal distances at 200–400nm was absent in heat-shocked nuclei with and without X irradiation (Figure 7D). pATM on the other hand showed a similar mean normalized frequency of signal distribution around 20 nm with a range of 10–80 nm in normo- and hyperthermia-treated cells, but lacked a mesoscale clustering of pATM signal tags (Figure 7E). The frequency of the spatial proximity at the nanoscale was more prominent in heat-treated cells, whereas the single molecule distance >100 nm followed a random distribution (Figure 7E). These results suggest that heat-shock alters the global chromatin organization that contributes to DSB repair.

## Discussion

Hyperthermia is of global concern, especially during hot environmental conditions that primarily select certain population groups who already face higher risks of heat-related death (Kumar and Singh, 2021; Medina-Ramon and Schwartz, 2007; Melvin et al., 2016; Navarro et al., 2017; Wobus et al., 2016; Zanobetti et al., 2012). At the cellular level, exposure to excess heat may induce cell death, which may follow DNA damage, but little is known as to how heat-shock impacts DNA damage repair pathways. Whereas hyperthermia does not directly induce DNA DSBs (Hunt et al., 2007), it may impact the DNA damage response, possibly through histone modifications that alter chromatin structure and cause impairment of repair protein recruitment to damaged DNA.

**Figure 8:**
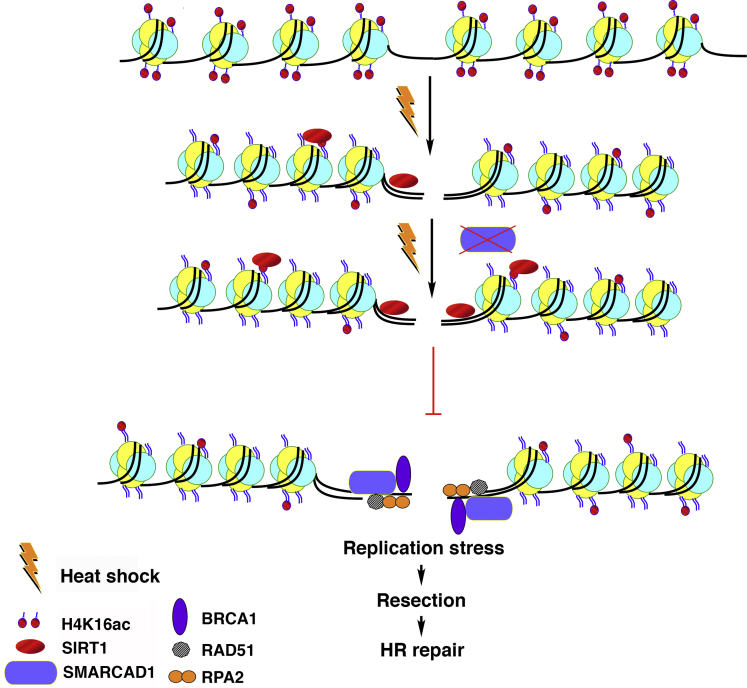
Heat-induced H4K16ac deacetylation impairs DNA resection compromising HR repair Hyperthermia induction leads to SIRT1-mediated deacetylation of H4K16ac. SIRT1 recruitment reduces the occupancy of SMARCAD1 to the sites of DSBs, thereby impairing DNA resection and causing HR repair defect.

Here we investigated patient cells, cell lines, and animals subjected to heat stress and detected that heat-shock-induced deacetylation of H4K16ac across different species and cell lines. As shown in the model (Figure 8), we demonstrated that heat-shock preferentially alters SIRT1 deacetylation of H4K16ac at gene-rich regions, a response that is instrumental in preventing the recruitment of SMARCAD1 onto DSBs and thereby compromising DNA end resection. Decreased H4K16ac levels have also been shown to affect the expression of mitochondrial uncoupling protein-2, huntingtin-interacting protein-1, and Notch-pathway genes; thus, H4K16ac levels are likely important in the response to ischemia and cell energy stress (Dmitriev and Papkovsky, 2015). We did not observe any significant heat-shock mediated alteration in MOF levels. Identifying SIRT1 as the prime candidate for maintaining the differential H4K16ac levels following hyperthermia treatment. Among the Sirtuins, SIRT1 significantly targeted the gene-rich regions. Silencing of SIRT1 in heat-shocked cells rescued deacetylation of H4K16ac, as compared with control cells exposed to hyperthermia. The reduction in H4K16ac by heat-shock has direct consequences in reprogramming chromatin-mediated transcription of gene-rich regions. This was directly evident by the heat-shock-mediated reduction of RNA Polymerase II occupancy at DSB sites.

H4K16ac marks active genes, whereas embryonic stem cell enhancers (Gupta et al., 2008; Horikoshi et al., 2013; Taylor et al., 2013) constrain the formation of higher order chromatin structures. This suggests that H4K16ac may force chromatin into a more open configuration and/or serve as a signal to facilitate the assembly of DDR components. We have recently reported that pre-existing H4K16ac levels directly impact DSB repair and that such acetylation is critical for DSB repair by the HR pathway (Horikoshi et al., 2019). On the other hand, the effect of hyperthermia on DSB repair by NHEJ was minimal as hyperthermia did not alter the proportion of uncut DNA relative to control cells. The depletion of Ku80 showed a similar decrease in the amount of uncut DNA irrespective of heat stress leading us to conclude that the NHEJ pathway was least impacted by heat-shock, whereas the HR pathway was significantly affected. The reduction in the frequency of DSB repair by HR correlated with reduced recruitment of HR-related proteins at the DSBs sites after heat-shock.

Hyperthermia impacts the recruitment of multiple HR-related proteins at DNA damage sites. Heat stress alone or in combination with CRISPR-induced site-specific DSBs caused enrichment of γ-H2AX in both gene-rich and gene-poor regions. However, heat-shock abrogated SMARCAD1, BRCA1, RAD51, RPA2 enrichment at DSBs. All HR-related protein factors examined increased DSB occupancy upon CRISPR-induction, and their occupancy was significantly reduced in cells treated with heat. Thus, heat-shock impairs the recruitment of repair proteins to DNA damage sites. Single molecule analysis showed that hyperthermia induced a global non-DSB-dependent phosphorylation of H2AX histone molecules with the γ-H2AX signal being organized in nano-clusters throughout the nucleus, whereas DSBs seeded by X-irradiation failed to induce further super clusters (microscopic foci) in the chromatin of heated nuclei, suggesting that this global γ-H2AX distribution is associated with impaired recruitment of DSB factors. Indeed, repeated particle irradiation has shown that 53BP1 is available in a limited nuclear pool that reduces further binding after a delayed of 53BP1 and Rad51 to DSB surrounding chromatin after a delayed second irradiation (Greubel et al., 2008), an effect that may play a role when massive γ-H2AX activation in the nucleus of heat-shocked cells leads to sequestering of HR factors. We also observed single molecule signals of active pATM foci formed at a lower rate than γ-H2AX signal tags/nucleus after heat-shock, but this may be owing to a increased loss of pATM molecules during the staining process, as ATM is sensitive to extraction variables (Noon et al., 2010), which may play a role in γ-H2AX-rich chromatin of heated cells. Still the number of pATM single molecule tags was increased by X-irradiation in control and heat-treated cells, indicating that ATM still responds to DSB induction in heat-treated chromatin.

Our report shows how hyperthermia impacts H4K16ac status and replication stress response. Moreover, it demonstrates that heat-induced deacetylation is SIRT1-dependent impacting chromatin dynamics and HR repair protein and SMARCAD1 recruitment at damage sites, and DNA resection to compromise the DDR. The depletion of chromatin modifying factor “SMARCAD1” resulted in hyperthermia-mediated sensitization to cell killing, increased sister chromatid exchanges (SCEs) as well as replication fork regression/restart defects. Taken together, the present study comprehensively elucidates physiologically the molecular consequences of heat stress by SIRT1-deacetylation of H4K16ac and recruitment of the chromatin remodeler protein SMARCAD1, leading to impaired DNA damage repair. These events appear widely conserved during evolution.

### Limitations of the study

In this study, we have elucidated the role of SIRT1-mediated H4K16ac deacetylation that impair the recruitment of DNA repair proteins involved in resection during hyperthermia. Interestingly, we show that SIRT1 function is negatively impacted by SMARCAD1, a member of SNF2 family of chromatin remodelers. Although the deacetylation of H4K16ac as a sequel to hyperthermia was confirmed in multiple organisms, like yeast, drosophila, mice, and human cell lines, and subsequently validated in hyperthermia patients, it has to be borne in mind that the detailed molecular mechanism could have variations across these systems. The fundamental molecular events were chased in cell-based systems through multiple approaches; however, the detailed insights were beyond the scope of this manuscript.

## STAR★Methods

### Key resources table

**Table:** 

REAGENT or RESOURCE	SOURCE	IDENTIFIER
**Antibodies**		

H3	Abcam	ab1791; RRID: AB_302613
H4	Abcam	ab10158; RRID: AB_296888
H4K16ac	Abcam	ab109463; RRID: AB_10858987
H4K8ac	Abcam	ab15823; RRID: AB_880455
SMARCAD1	Santacruz Biotech	Sc-162233; RRID: AB_2191867
SMARCAD1	Novus Bio	H00056916-BO1P; RRID: AB_2191869
MRE11	Gene Tex	70,212; RRID: AB_372398
MRE11	Novus Bio	NB100-142; RRID: AB_10077796
γ-H2AX	Millipore	JBW301; RRID: AB_309864
	-	-
RAD51	Abcam	Ab213; RRID: AB_302856
BRCA1	Santacruz Biotech	sc-642; RRID: AB_630944
53-BP1	Santacruz Biotech	sc-22760; RRID: AB_2256326
RPA 70	Abcam	ab 79,398; RRID: AB_1603759
Flag M5	Sigma	F-4042; RRID: AB_439686
CldU	Bioscience	347,580; RRID: AB_10015219
IdU	Novus Bio	NB-500-169; RRID: AB_10002608
Tubulin	Santacruz Biotech	sc-5286; RRID: AB_628411
GAPDH	Cell Signaling	8884S; RRID: AB_11129865
β-actin	Santa Cruz Biotechnology	Sc-8432; RRID: AB_626630

**Bacterial and virus strains**		

*E.coli.* DH5α competent cells	Lab generated	N/A

**Biological samples**		

Peripheral blood mononuclear cells from human subjects (control cohort and hyperthermia patients)	Department of Medical Oncology and Department of Medicine at All India Institute of Medical Sciences (AIIMS) New Delhi India (IEC-363/08.05.2020,RP-06/2020)	N/A

**Chemicals, peptides, and recombinant proteins**		

Protease inhibitor cocktail complete EDTA free	Roche	4693116001
Aprotinin	Sigma	A1153
Leupeptin	Sigma	103,476-89-7
PMSF	Sigma	329-98-6
Sodium Orthovanadate	Sigma	13,721-39-6
Sodium Fluoride	Sigma	7681-49-4
Sodium Deoxycholate	Sigma	30,970
Nonidet P-40	Thermo Fischer	85,124
Flag-M2 agarose beads	Sigma	A2220
Magna Protein A/G beads	Millipore	16–663
Propidium iodide	Sigma	P4864
Hydroxyurea	Sigma Aldrich	C9911
BrdU	Sigma Aldrich	B5002
CldU	Sigma Aldrich	C6891
IdU	Sigma Aldrich	I7125

**Critical commercial assays**		

Nucleic Acid Extraction Kit	Thermo Fischer	4452222
cDNA synthesis kit	Thermo Fischer	K16315

**Experimental models: Cell lines**		

HCT116 human cell line	ATCC	CCL-247; RRID: CVCL_0291
HEK293 human cell line	ATCC	CR-L-1573; RRID: CVCL_0045
Hela human cell line	ATCC	CRL-12401; RRID: CVCL_W341
H-1299 human cell line	ATCC	CRL-5803; RRID: CVCL_0060
U2OS HA-ER AsiSI cell line	ATCC	CRL-3455; RRID: CVCL_B0A7
CHO	ATCC	CRL-3440; RRID: CVCL_A4AR

**Experimental models: Organisms/strains**		

Drosophila Canton S wild-type	ATCC	CRL-1963
Nude nu/nu female athymic mice	Charles River Laboratory, Wilmington	N/A
*Schizosaccharomyces pombe*	ATCC	64,404

**Oligonucleotides**		

DSB1-335 bp-fw GAATCGGATGTATGCGACTGATC	This Paper	N/A
DSB1-335 bp-Rev TTCCAAAGTTATTCCAACCCGAT	This Paper	N/A
DSB1-335 bp- Probe-6FAM CACAGCTTGCCCATCCTTGCAAACC-TAMRA	This Paper	N/A
DSB1-1618 bp-fw TGAGGAGGTGACATTAGAACTCAGA	This Paper	N/A
DSB1-1618 bp-Rev AGGACTCACTTACACGGCCTTT	This Paper	N/A
DSB1-1618 bp- Probe-6FAM TTGCAAGGCTGCTTCCTTACCATTCAA-TAMRA	This Paper	N/A
DSB1-3500 bp-fw TCCTAGCCAGATAATAATAGCTATACAAACA	This Paper	N/A
DSB1-3500 bp-Rev TGAATAGACAGACAACAGATAAATGAGACA	This Paper	N/A
DSB1-3500 bp- Probe-6FAM ACCCTGATCAGCCTTTCCATGGGTTAAG-TAMRA	This Paper	N/A

**Recombinant DNA**		

HA–ER *Asi*SI plasmid	Tanya T Paull, University of Texas, Austin	N/A
**Software and algorithms**		
Primer3	http://primer3.ut.ee/	N/A
ImageJ	https://imagej.nih.gov/ij/index.html	N/A

### Resource availability

#### Lead contact

Further information and requests for resources and reagents should be directed to and will be fulfilled by the lead contact, Dr. Tej K. Pandita (tpandita@tamu.edu).

#### Material availability

The plasmids, yeast strains, and cell lines generated from this study will be available upon material transfer agreement (MTA) completion. All reagents including antibodies, siRNA, and plasmids can be found in the key resources table.

### Experimental model and subject details

#### Human subjects

For this prospective study which was conducted in association with Department of Medical Oncology and Department of Medicine at All India Institute of Medical Sciences (AIIMS) New Delhi India, individuals enrolled were suffering from high grade fever between July to December 2020 having temperature near (∼40.5°C). Demographic and epidemiological details of all patients were also collected. The controls included who were not suffering from any fever (37°C or less) at time of sample collection or 3 months prior to sample collection. The study was approved by AIIMS institutional ethics committee (AIIMS IEC) bearing approval no IEC-363/08.05.2020, RP-06/2020 and written informed consent was obtained from all the subjects including patients and controls.

#### Mice

The heating of tumor cells in nude nu/nu mice was performed following approved protocols as described previously (Locke et al., 2005). Nude nu/nu female athymic mice were obtained from Charles River Laboratory, Wilmington, MA and inoculated at age 12–16 weeks old. Exponentially growing HeLa cells were resuspended in Dulbecco’s Modified Eagles Media (DMEM) (Sigma, St. Louis, MO) in 30% matrigel solution (BD Biosciences, Bedford, MA) and two million cells were injected via subcutaneous injection bilaterally into the proximal thighs. Mice were inoculated at two different sites to grow two tumors to an average size of 350 mm^3^. Six mice bearing two tumors for each condition were compared with a similar number of untreated controls using either a water bath or ultrasound treatment delivery. Mice were obtained from Jackson Laboratories, USA and experiments were performed after the approval of Animal Studies Committee of Washington University School of Medicine, Saint Louis, MO, USA.

#### Drosophila

Canton S wild-type flies were raised in standard fly food media at 25°C as described previously (Bhadra et al., 2012). Third-instar larvae were given heat-shock at 37°C for 1 and 2h and maintained at 25°C for 30 min prior to dissection. Control larvae did not receive heat-shock.

#### Cell lines

HeLa, HEK 293, H-1299, CHO, HCT116 cell lines (ATCC) were grown in DMEM (Sigma) and 10% fetal calf serum (Sigma) supplemented with 1% penicillin/streptomycin. The U2OS HA-ER *Asi*SI cell line was maintained in DMEM (Sigma) and 10% fetal bovine serum (Sigma) supplemented with 1% penicillin/streptomycin and 1 μg/mL puromycin. The cells were heated at 43°C for different time points from 5 to 60 min followed by 60 min recovery.

#### Schizosaccharomyces pombe

Yeast strain BY4741 used in the study was provided by Prof. Jacques Cote. Overnight primary culture yeast cells grown at 30 °C were inoculated in four falcon tubes each containing 20 mL YPD media and grown up to an (OD_600_) = 1. The tubes were labeled 0 h, 30 min, 1 h and 2 h, each representing the time point at which cells were subjected to heat stress from physiological growth conditions (30 °C) to heat shock conditions (40 °C). Cells were pelleted down and lysed with bead beating in RIPA buffer contacting 50 mM HEPES, 2mM EDTA, 1% v/v Triton X-100, 250 mM NaCl, 0.1% v/v Sodium deoxycholate, 0.1% SDS and Protease Inhibitor Cocktail. Supernatant was collected and followed by protein estimation by Bradford method. Equal amount of protein (30 μg) were loaded onto 15% polyacrylamide gel followed by western blot using H4K16 acetyl (active motif, Cat No.39167) and H3 antibodies (abcam, AB1791).

### Method details

#### Clonogenic assay

HeLa/H1299/HCT116/CHO/HEK293 cells were used for clonogenic assays. siRNA transfection was done in 2×10^5^ cells/mL using Nucleofector and treated as required. Cells were then trypsinized and transferred to T-25 flasks. For hyperthermia experiments the T-25 flasks were incubated in a 43°C water-bath for 30 min and then exposed to various doses of ionizing radiation (IR). Flasks were incubated for 10-14 days. Each individual group was processed in triplicate and normalized to untreated controls. The survival graphs show combined data from at least three independent experiments, and error bars show standard error.

#### PBMCs (peripheral blood mononuclear cells) isolation from human blood

4-6 mL of blood was collected in the EDTA vial. It was diluted with PBS (approximately in 1:1 ratio). Ficoll gradient was generated in a falcon tube (1:2 Ficoll solution: blood sample). The blood was added in to the falcon tube using dropper slowly over the layer of ficoll by keeping the falcon tube tilted at 30 angle. Centrifugation was done at 2000 rpm for 30 min. A ficoll gradient was formed and buffy coat layer was collected in the separate falcon. To red blood cells (RBCs) cell lysis buffer was added to lyse the RBCs at room temp for 10-15 min following which centrifugation was done at 12,000 rpm for 10 min. PBMCs were pelleted down and washed with PBS. Centrifugation was done at 12,000 rpm for 10 min and PBMCs were collected. PBMCs were further processed for western blotting.

#### *In vivo* hyperthermia treatment

Mice were anesthetized via intraperitoneal injection of ketamine/xylazine cocktail (1 mg/kg) before heat-shock and were placed on a slatted metal platform suspended over the water bath with a tumor bearing leg positioned through a slat and immersed into the water. For control, the contralateral tumors bearing leg was suspended above the platform and to prevent contact with the water bath. Tumor temperature was monitored using a metal thermocouple probe (type T, Ella-CS, Czech Republic), which was placed in the middle of the tumor at central axis to confirm a temperature of 43°C. Water bath temperature was controlled with a computerized heating device with a variance of less than 0.2°C (Locke et al., 2005; Singh et al., 2004). The study was performed with institutional approval from the Animal Studies Committee of Washington University School of Medicine, Saint Louis, MO, USA.

For heating with ultrasound, anesthetized mice were placed on the small animal ultrasound system (SAHUS) device with tumors aligned to the 5 MHz ultrasound transducers and tumor temperature of 43°C monitored with a single needle thermocouple placed in the center of the tumor as described (Locke et al., 2005; Singh et al., 2004).

Tumors were snap frozen in liquid nitrogen. The tissue samples were washed twice with 1X PBS buffer and incubated for 15 min in 200 mL of cell lysis buffer (Sigma, St. Louis, MO). The tissue was then homogenized using a chilled micro-homogenizer, centrifuged and supernatant stored in liquid nitrogen until use. Western blots with specific antibodies were performed as described previously (Hunt et al., 2004).

To study the effect of hyperthermia on histone H4K16acetylation *in vivo*, HeLa xenotransplants were examined. Established tumors of HeLa xenotransplants of about 750 mm^3^ were treated for hyperthermia in a water bath set at 43°C for 30 min (Locke et al., 2005). After hyperthermia, tumor cells were frozen in liquid nitrogen and protein extraction performed as described (Locke et al., 2005).

#### *Drosophila* larval salivary gland and fat body immunostaining

*Drosophila* larvae were dissected in phosphate-buffered saline (PBS) and fixed for 20 min in 4% p-formaldehyde. The larvae were washed with PBS and incubated in PBS containing 0.3% Triton X-100 and 10% NGS (normal goat serum) for 2-3h. The larvae were incubated with anti-H4K16ac antibody (Abcam, 1:200 dilution in PBS containing 0.3% Triton-X 100 and 10% NGS) overnight at 4°C with shaking. The larvae were washed with PBS containing 0.3% Triton X-100 for 20 min thrice and incubated with secondary antibody conjugated with fluorophore (Alexa dyes, Invitrogen, 1:400 dilution in PBS containing 0.3% Triton X-100 and 10% NGS). Thereafter the larvae were washed for 20 min thrice, the salivary gland and fat body were dissected and mounted on antifade containing DAPI. Fluorescent images were acquired using the Zeiss LSM880 confocal microscope.

#### Drosophila polytene chromosome immunostaining

Third instar larvae were heat-shocked at 37°C for 1.5 h. Around 3-4 salivary glands were dissected from heat-shocked and control larvae. The polytene chromosome preparation were prepared for male and female salivary gland as described previously (Cai et al., 2010). The polytene squash preparations were immunostained with anti-H4K16Ac antibody and counterstained with Hoechst 33,258 (Molecular probes) as described previously (Johansen et al., 2009). The polytene chromosomes were imaged using the Zeiss LSM880 confocal microscope.

#### Direct-repeat green fluorescent protein (DR-GFP) based HR assay

DR-GFP based assay was performed as described (Pierce et al., 1999). Stably expressing DR-GFP cassette was introduced into H-1299 cells. HR assay was monitored as the percentage of GFP positive cells by flow cytometry.

#### DNA end resection assay

The U2OS cells with stably expressing HA-ER-AsiSI were generated as described (Zhou et al., 2014). We quantified ss-DNA using an HA-ER-AsiSi system in which AsiSI enzyme is fused to an Estradiol Response Element. Upon addition of 4-hydroxy-Tamoxifen (4-OHT), the AsiSI enzyme goes to the nucleus to generate double strand break at the sequence specific AsiSI (GCGATCGC) sites. Genomic DNA was extracted and ss DNA in Chromosome 1 detected by qPCR using specific primers as mentioned in the key resources table.

#### Western blotting and immunoprecipitation

The cell lysate was prepared in ice-cold lysis buffer (50 mM Tris-HCl, pH 7.4, 200 mM sodium chloride, 2 mM EDTA, 0.5% Na-deoxycholate, 1% NP-40). Protease inhibitors, Aprotinin (0.5 μg/mL), Leupeptin (0.5 μg/mL), PMSF (1mM) Sodium orthovanadate (0.2mM), Sodium Fluoride (50mM) were added fresh. Samples were processed for SDS–PAGE and western blotting by addition of 5X loading buffer and boiling. For immunoprecipitation, cell lysates were incubated with the required antibody (2 μg) at 4°C overnight. Protein A/G plus agarose beads were added next day and incubated at 4°C for 1 h. The agarose beads were then washed 3-4 times with NETN buffer (200mM Tris-HCl,100mM NaCl, 1mM EDTA, 10% Glycerol, 0.1% NP-40 and protease inhibitors).

*Schizosaccharomyces pombe* were grown at optimal temperature (32°C) to an OD of 1–1.5 and then shifted to 40°C for the specified time points. Cells were pelleted down and lysed using the bead beating method. Samples were analyzed for H4K16ac and H4 levels by Western blotting. The band intensity of the western blots was quantified using ImgeJ software and was normalized to loading control α-tubulin or α-actin. For histone H4K16ac or H4K8ac, normalization was done with histone H4.

#### Chromosome aberration and sister chromatid exchange (SCE)

Metaphase chromosomes were prepared by using the standard procedures as described previously (Pandita et al., 1995, 2006; Hunt et al., 2007). The procedure for heat treatment followed by irradiation was the same as described previously (Hunt et al., 2007). Giemsa-stained chromosomes from metaphase spreads were analyzed for aberrations. For scoring chromosome aberrations, 100 metaphases for each experiment were scored and each experiment was repeated three to four times.

Sister chromatid exchanges were studies as described previously (Pandita, 1983). Cell in culture were incubated in presence of BrdU (10 mg/mL) for two rounds of replication. Exposure to heat was as reported previously (Hunt et al., 2007). Metaphases were collected during the last 3h with colcemid at 0.30/μg/mL. Flame-dry preparations were made and stained by a modified fluorescence plus giemsa technique as described earlier (Pandita, 1983; Chakraborty et al., 2018). For scoring SCE frequency, 100 metaphases of second cycle from each experiment were studied. Each experiment was repeated three times.

#### DNA fiber assay

DNA fiber analysis is done as described (Tuduri et al., 2010) with modifications, first using the nucleotide analog CldU (Green) and subsequently treatment with DNA damaging agent hydroxyurea (2mM for 2h) before release. The next pulse label was done using a second nucleotide analog IdU (red). At the end of the second labeling, cells were lysed and the DNA is spread onto glass slides. After staining the DNA with fluorophore-coupled antibodies against the halogenated nucleotides as indicated by the colors, the fibers were visualized by fluorescence microscopy.

#### Chromatin immunoprecipitation assay (ChIP)

Chromatin immunoprecipitation assay was done in RPAB and Ch1 I-Sec1 cell lines. The RPA B cell line was generated by introduction of I-sce1 (18 bp TAGGGATAACAGGGTAAT) within the second intron of the *RPA* gene in Chromosome1 (highly transcribing region) utilizing CRISPER system in H1299 cells as described (Horikoshi et al., 2019). Ch1I-Sec1 cell line was established by introducing I-Sec1 site in the non-transcribing region of Chromosome 1. I-Sec1 plasmid was transfected in these two cell lines (2x10^6^ cells per sample) 24h before processing for ChIP assay. Crosslinking was done with 1% Formaldehyde on a rocking platform for 10 min at room temperature and stopped by addition of 1.37M Glycine (100 μL/mL) for 5 min. The plates were washed three times with ice-cold PBS (containing protease inhibitors). The cell pellet was collected and resuspended in 300μL of sonication buffer (50mM Tris-HCl pH 8.0, 10mM EDTA, 1% SDS and protease inhibitors). Sheared chromatin was centrifuged at 20,000g for 10min at 4°C. Supernatant was collected and diluted (1:10) with ChIP dilution buffer (16.7mM Tris-HCl, pH 8.0,167mM NaCl, 1.2mM EDTA, 1.1% Triton X-100, 0.1% SDS). Diluted chromatin was incubated with 2μg of antibody and Magna ChIP Protein A/G beads (Millipore) overnight at 4°C on a rocking platform. Beads were washed with wash buffer I (20mM Tris-HCl, pH 8.0,150mM NaCl, 1.2mM EDTA, 1% Triton X-100, 0.1% SDS + PI),wash buffer (20mM Tris-HCl, pH 8.0, 500mM NaCl, 1.2mM EDTA, 1% Triton X-100, 0.1% SDS + PI), wash buffer III (10mM Tris-HCl, pH 8.0, 1mM EDTA, 1% Sodium deoxycholate, 1% NP-40, 0.25M Lithium Chloride). Samples were then washed twice with TE buffer. ChIP samples were eluted with 200ul freshly made warm (65°C) ChIP elution buffer (0.1mM NaHCO3, 0.01% SDS, dH2O). Samples were decrosslinked with 8ul of 5M NaCl at 65°C overnight. DNA was purified using a company provided kit (Catalog number: 4,452,222).

ChIP assays were performed as per the standard protocol (Horikoshi et al., 2019). In brief, 1% formaldehyde was used to cross-link proteins to the DNA at room temperature for 10 min. This reaction was then immediately stopped by adding 125mM Glycine to the media and incubated for 10 min. This was followed up by the cell lysis in cell lysis buffer (5 mM PIPES(pH 8.0), 85 mM KCl, 0.5% Nonidet P-40, 1X protease inhibitor cocktail) for isolation of nuclei. Isolated nuclei were then resuspended in nuclei lysis buffer (50 mM Tris-HCl (pH 8.0), 10 mM EDTA, 1% SDS, 1Xprotease inhibitor cocktail). After the lysates were sonicated and pre-cleared, chromatin was immuno-precipitated with anti-FLAG M2 Agarose bead overnight at 4 °C. Then the beads were washed with buffers in the following order: radio-immune precipitation assay buffer(50 mM Tris-HCl, pH 8.0, 150 mM NaCl, 0.1% SDS, 0.5% sodium deoxycholate, 1% Nonidet P-40, 1 mM EDTA), high-salt buffer(50 mM Tris-HCl, pH 8.0, 500 mM NaCl, 0.1% SDS, 0.5% sodium deoxycholate, 1% Nonidet P-40, 1 mM EDTA), LiCl buffer(50 mM Tris-HCl, pH 8.0, 250 mM LiCl, 0.5% sodium deoxycholate, 1% Nonidet P-40, 1 mM EDTA), and Tris-EDTA(10 mM Tris-HCl, pH 8.0, 1 mM EDTA). This was then followed with RNase A and proteinase K treatment, after which the beads were kept at 65 °C overnight for decrosslinking. The next day Phenol/chloro-form extraction followed with ethanol precipitation was done and DNA was pelleted down. The DNA pellet was then dissolved in water and processed for qPCR analysis using gene-specific primers for Chr1A, Chr1B, Chr17A and Chr5B as described (Horikoshi et al., 2019).

#### Calculation of fold change

Fold change calculations were performed using the comparative change in the IP sample with respect to the IgG sample, and in our case, the flag overexpressed sample.

For this we first calculated the IgG value (A)

(Equation 1) IgGValue(A)=AverageQRTvalueoftheIgG−AdjustedinputvalueoftheIgG

which we got after subtracting the average value of QRT of IgG from the adjusted input for the same.

For calculating the IP value (B), and similar equation to Equation 1 was used:

(Equation 2) IPvalue(B)=AverageQRTvalueoftheIP−AdjustedinputvalueoftheIP

We then subtracted the value of A from B to obtain ΔCt value

ΔCt=B−A

From the ΔCt fold change or the ΔΔCt were calculated fold change = 2^−ΔCt^

The fold change values were normalized by taking maximum fold change as one for each set followed by calculation of the average value from the replicate sets. The error bars in the graph reflect Standard Deviations (SD) between the replicate sets.

#### Statistical analysis

An unpaired two-tailed Student’s *t* test was performed and Statistical significance were determined using the Holm-Sidak method (alpha = 0.05) using the GraphPad Prism software to assign the significant differences between the groups. Significant differences were taken when ∗p ≤ 0.05, ∗∗p ≤ 0.01, ∗∗p ≤ 0.001, and ∗∗∗p ≤ 0.0001.

#### Single molecule localization microscopy (SMLM)

SMLM was used to record and map the γ-H2AX and pATM fluorescent antibody tags. The different treatments of HeLa cells grown on slides were: 1) no treatment (37°C, control) and 1 Gy X-ray irradiation of control cells; 2) pre-heating of cells for 30 min at 43°C + sham irradiation; HT + 1Gy X-IR. After the sham or 43°C treatment, cells were fixed 30 min after culture at 37°C (to allow for γ-H2AX focus formation after IR). The cells were then stained for phosphorylated H2AX (γ-H2AX) and activated ATM (p-S1981-ATM). Proteins were labeled using immunofluorescence staining with secondary Abs labeled with Cy3 and Cy5 fluorophores (Scherthan et al., 2019). SMLM was used to record and map the γ-H2AX and pATM fluorescent antibody tags. We recorded all fluorophore blinking events (single molecule signals) from γ-H2AX and pATM in n = 20 cells for each sample. For each plot we only considered the signals detected inside a DAPI-defined nuclear mask.

## Data Availability

• Microscopy data and western blot data are reported in this paper, the raw data/images will be shared by the lead contact upon request • The original ChIP information is available in published paper (https://doi.org/10.1038/s42003-019-0498-z.) • Any additional information required to reanalyze the data reported in this paper is available from the lead contact upon request.
