# Supplementary material for: Heat-induced SIRT1-mediated H4K16ac deacetylation impairs resection and SMARCAD1 recruitment to double strand breaks
Source: iScience. 2022 Mar 23;25(4):104142. doi: 10.1016/j.isci.2022.104142 (PMC9010620; doi:10.1016/j.isci.2022.104142)
Supplement: Document S1. Figures S1–S7 [file mmc1.pdf]

iScience, Volume *25*

Supplemental information

Heat-induced SIRT1-mediated H4K16ac deacetylation impairs resection and SMARCAD1 recruitment to double strand breaks

Sharmistha Chakraborty, Mayank Singh, Raj K. Pandita, Vipin Singh, Calvin S.C. Lo, Fransisca Leonard, Nobuo Horikoshi, Eduardo G. Moros, Deblina Guha, Clayton R. Hunt, Eric Chau, Kazi M. Ahmed, Prayas Sethi, Vijaya Charaka, Biana Godin, Kalpana Makhijani, Harry Scherthan, Jeanette Deck, Michael Hausmann, Arjamand Mushtaq, Mohammad Altaf, Kenneth S. Ramos, Krishna M. Bhat, Nitika Taneja, Chandrima Das, and Tej K. Pandita

Supplemental Information

Heat-induced SIRT1-mediated H4K16ac deacetylation impairs resection and SMARCAD1 recruitment to double strand breaks

Sharmistha Chakraborty[1,2a], Mayank Singh[2,3a], Raj K. Pandita[1,2,4,5a], Vipin Singh[6,7a], Calvin S.C. Lo[8], Fransisca Leonard[1], Nobuo Horikoshi[1,2,5], Eduardo G. Moros[5,9], Deblina Guha[7], Clayton R. Hunt[1,2,5], Kalpana Makhijani[7], Eric Chau[1], Kazi M. Ahmed[1], Prayas Sethi[3], Vijaya Charaka[1], Biana Godin[1], Kalpana Makhijani[10], Harry Scherthan[11], Jeanette Deck[12], Michael Hausmann[12], Arjamand Mushtaq[13], Mohammad Altaf[13], Kenneth S. Ramos[14], Krishna M. Bhat[10], Nitika Taneja[8], Chandrima Das[6,7] and Tej K. Pandita[1,2,4,5,14]

[1]Department of Radiation Oncology, Houston Methodist Research Institute, Houston, TX, 77030, USA; [2]Department of Radiation Oncology, University of Texas Southwestern Medical Centre, Dallas, TX; [3]Department of Medical Oncology, All India Institute of Medical Sciences, New Delhi 110029, India; [4]Department of Molecular and Cellular Biology, Baylor College of Medicine, Houston TX 77030, USA; [5]Departments of Radiation Oncology, Washington University St Louis, MO; [6]Biophysics & Structural Genomics Division Saha Institute of Nuclear Physics, Bidhan Nagar, Kolkata, West Bengal, 700064, India; [7]Homi Bhaba National Institute, Mumbai, India; [8]Department of Molecular Genetics, Erasmus MC Cancer Institute, Erasmus University Medical Center, 3000 CA Rotterdam, The Netherlands; [9]Departments of Radiation Oncology, Moffitt Cancer Center Tampa, FL 33612, USA; [10]Department of Molecular Medicine, University of South Florida, Tampa, FL, 33612, USA; [11]Bundeswehr Institute of Radiobiology Affiliated to the University of Ulm, Neuherbergstr. 11, D-80937 Munich, Germany; [12]Kirchhoff-Institute for Physics, Heidelberg University, Im Neuenheimer Feld 227, 69120 Heidelberg, Germany; [13]Department of Biotechnology, University of Kashmir, Srinagar, Jammu and Kashmir, 190006, India; [14]Center for Genomics and Precision Medicine, Texas A&M College of Medicine, Houston, Texas, USA.

Supplementary Figure Legends

Supplementary Figure 1 : Heat shock reduces cellular H4K16ac levels. (Data related to Figure 1). (**a,b,c**) Western blot from cell extracts of HeLa cells: (**a**) Showing H4K16ac at different temperature exposed for 30 min. (**b**) H4K16ac levels after exposure for different times at 43°C. (**c**) H4K8ac levels upon heat treatment of 43°C for 30 and 60 mins. (**d**) Immunofluorescence with H4K16ac antibody upon similar heat treatment in HeLa cells (Scale bar is 10 μm). (**e**) H4K16ac staining, following heat shock treatment, from fat body and salivary gland of male and female *Drosophila* (Scale bar is 100 μm). **(f, g)** Western blot showing effect of heat-shock for 15, 30 and 60 min on H4K16ac levels in *Schizosaccharomyces pombe* analyzed (**f**) and quantified (**g**).

Supplementary Figure 2: Heat shock effects on cell survival and levels of H4K16ac and MOF levels (Data related to Figure 2**).** (**a, b**) Survival assays are performed upon heat shock, IR and a combination of the two in H1299, 293, HCT116 (**a**) and HeLa, U2OS and CHO (**b**) cells. Heat treatment did not alter the overall survival. However, compared to IR alone, a combination of heat and IR caused a further reduction in cell survival in all these cell lines. (**c**) Western blot showing knockdown of MOF with specific siRNA. (**d**) A combination of IR and heat in MOF-silenced background led to a significant reduction in cell survival as compared to IR alone or in combination with MOF siRNA. (**e**) Heat alone does not cause any significant chromosome aberration. An alteration in chromosome aberration during a combination of Heat and IR treatment was found to be more robust as compared to IR alone in H1299, 293 and HCT116 cells.

Supplementary Figure 3: Heat-shock impacts SIRTI-mediated H4K16ac deacetylation and DSB induction (Data related to Figure 3**).** (**a**) The expression of endogenous SIRT1, SIRT2 and SIRT4 upon 30 mins and 60 min heat treatment. No significant change in expression of SIRT1/2/4 was observed. (**b**) FLAG-conjugated SIRT1/SIRT2/SIRT4 were ectopically expressed in HeLa cells followed by similar heat treatment. Marginal changes in the expression of these FLAG-conjugates could be detected upon hyperthermia induction. (**c-f**) ChIP assays following transfection with FLAG- conjugated SIRT1/SIRT2/SIRT4 were performed. The enrichment of SIRT1 was most prominent in gene-rich region (Chr1A or Chr17A) following heat treatment (**c**). SIRT2 shows a reduced occupancy in Chr1A and a concomitant increase in Chr1B and Chr17A upon hyperthermia induction (**d**). SIRT4 shows reduced enrichment in Chr1A and an increased recruitment in Chr17A upon induction of heat stress (**e**). No significant difference in the recruitment of SIRT6 could be seen in gene-rich/gene-poor regions upon heat shock (**f**).

Supplementary Figure 4: Impact of heat shock on recruitment of DSB repair factors in gene-rich regions. (Data related to Figure 4**). (** (**a, b**) The HR and NHEJ frequency is calculated in different sites (A and B) of Chromosome 1, 5, 17. There is reduction in HR frequency following heat treatment in Site A (**a**). Invoking site-specific DNA damage by I-SceI in combination with heat, showed almost similar NHEJ frequency, indicating this pathway is not affected (**b**). As a control, Ligase IV silencing has been done which caused a significant reduction in NHEJ

frequency (**b**). (**c, d**) Western blot showing no significant alteration in Rad51 (**c**), SMARCAD1 and MOF (**d**) could be observed upon heat-shock.

Supplementary Figure 5: Replication stress by heat shock as measured by DNA fiber tract length (Data related to Figure 5)**.** (**a**) Representative images of DNA fibers showing CldU green and IdU red labeling with and without heat and HU treatment in cells with and without SMARCAD1 depletion. (**b**) Graph showing a significant reduction in frequency of CldU + IdU fibers in heat shocked cells. (**c**) An increase in stalled fork percentage in heat shocked cells. (**d**) A reduction in new replication origins in heat shocked cells (H1299). (**e**) IdU tract length of CldU + IdU tract did not show a change when treated with heat, HU or heat and HU treatment together after DNA synthesis. (**f**) MOF depletion enhanced the significant decrease in the frequency of CldU + IdU fibers. MOF depleted cells treated with heat or HU had higher decrease in the frequency of CldU + IdU fiber as compared to cells with MOF treated with heat shock or HU or both.

Supplementary Figure 6: SMARCAD1 depletion impacts SIRT1 recruitment (Data related to Figure 6)**.** (**a**) Western blot showing depletion of SMARCAD1 by specific siRNA at different time points after transfection. **(b)** Western blot showing depletion of SMARCAL1 by specific siRNA at different times points after transfection. **(c)** Depletion of SMARCAL1 has no impacts on the heat shock induced recruitment of SIRT1. (**d, e**) Percentage of stalled replication forks and new origins of replication was measured upon heat treatment in SMARCAD1 siRNA background in presence or absence of HU. HU treatment caused maximum % of stalled replication forks upon heat treatment in a SMARCAD1 knockdown

background (**d**). Firing of new origins of replication was most significant upon HU treatment in SMARCAD1 siRNA background (**e**). (**f**) Scheme of DNA resection assays using AsiSI system. (**g, h**) DNA damage foci marked by γ-H2AX was found to be significantly increased upon combining heat and post-irradiation (30-240 min) in SMARCAD1 silenced condition. Representative confocal images of γ-H2AX foci upon heat and post-irradiation (120 and 240 min) treatment in a SMARCAD1 knockdown background (**g**) (Scale bar 10 μm). SMARCAD1 silencing showed a significant reduction in γ-H2AX puncta formation as compared to heat and IR exposure (240 min recovery) alone. Graphic showing the quantitative representation of γ-H2AX in cells with and without depletion of SMARCAD1, with and without treatment of heat shock or irradiation. Note the significant differences in residual γ-H2AX foci seen in cells depleted with SMARCAD1, treated with heat shock and irradiation (**h**).

Supplementary Figure 7: Heat-shock impairs cluster formation of repairosome foci (Data related to Figure 7)**.** **(a)** Quantitative single molecule localization (SML) analysis of pATM signal tags. Images of nuclei showing pATM single molecule signals (red dots) and their clustering (light blue areas) in the differently treated nuclei (Scale bar is 1 μm). (**b**) Boxplots showing the average number of single molecule signal events for pATM per nucleus. Irradiation of normothermia exposed cells (0 Gy, 37°C) increases the average frequency of SML pATM signals per nucleus. Hyperthermia (43°C) reduced the no. of pATM SML tags per nucleus, which is likely due to protein loss during extraction (Greubel et al., 2008) of heat-exposed cells, while irradiation of HT-treated cells led to a

doubling of the average signal no. per nucleus. (**c**) Cluster analysis of pATM signals with the parameters $R_{cluster} = \leq 200nm$ and $N_{min} = 10$ shows a similar behavior as for the frequencies in the different conditions noted in A. Again, 1 Gy X-IR induces a ~33% increase in numbers of ATM aggregates, in agreement with IR inducing ATM phosphorylation. Heat-treated cells display fewer clusters of pATM signals but 1 Gy X-IR again induces a ~40% increase in clustering.

Supp Figure 1

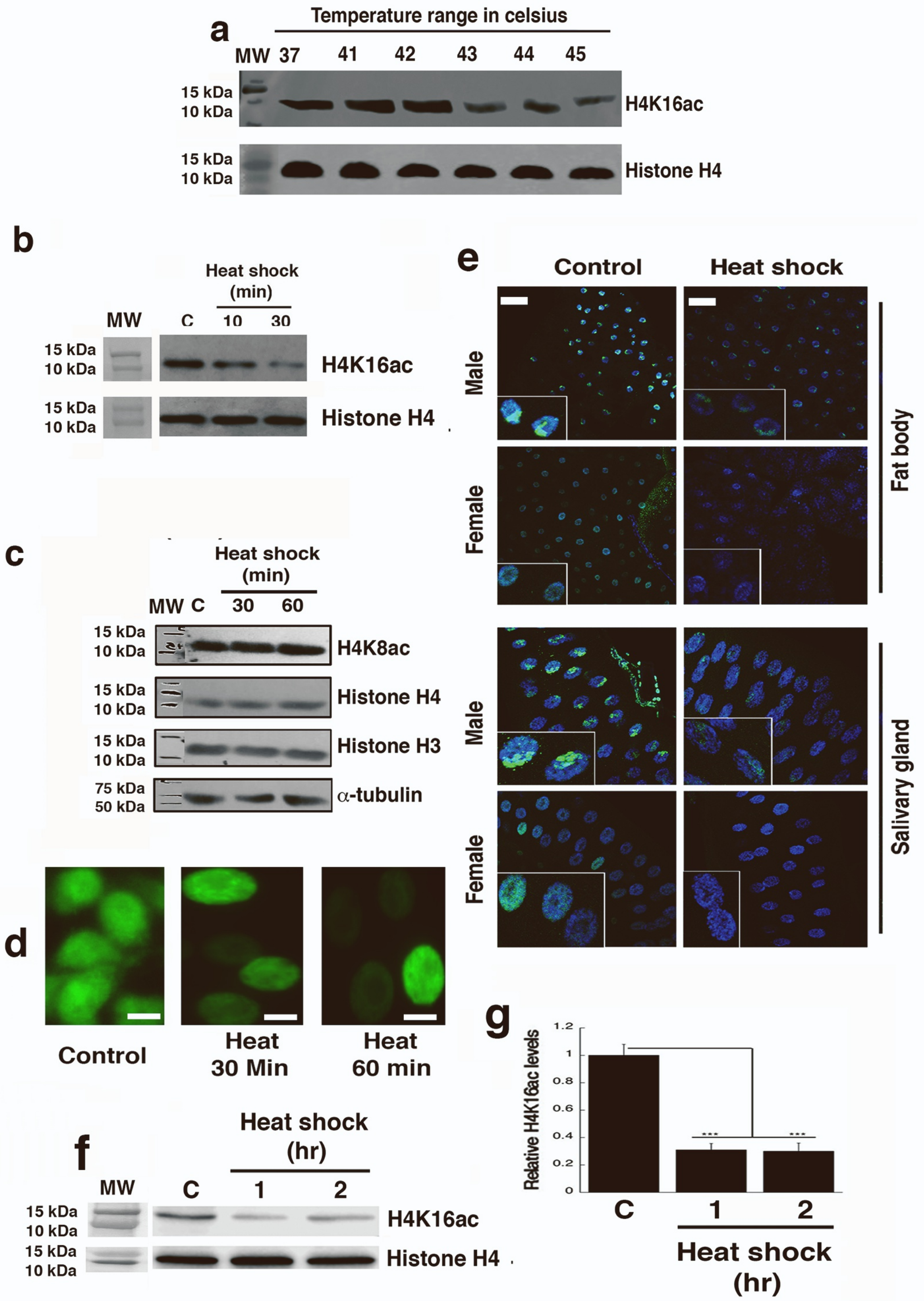

Supp Fig. 2

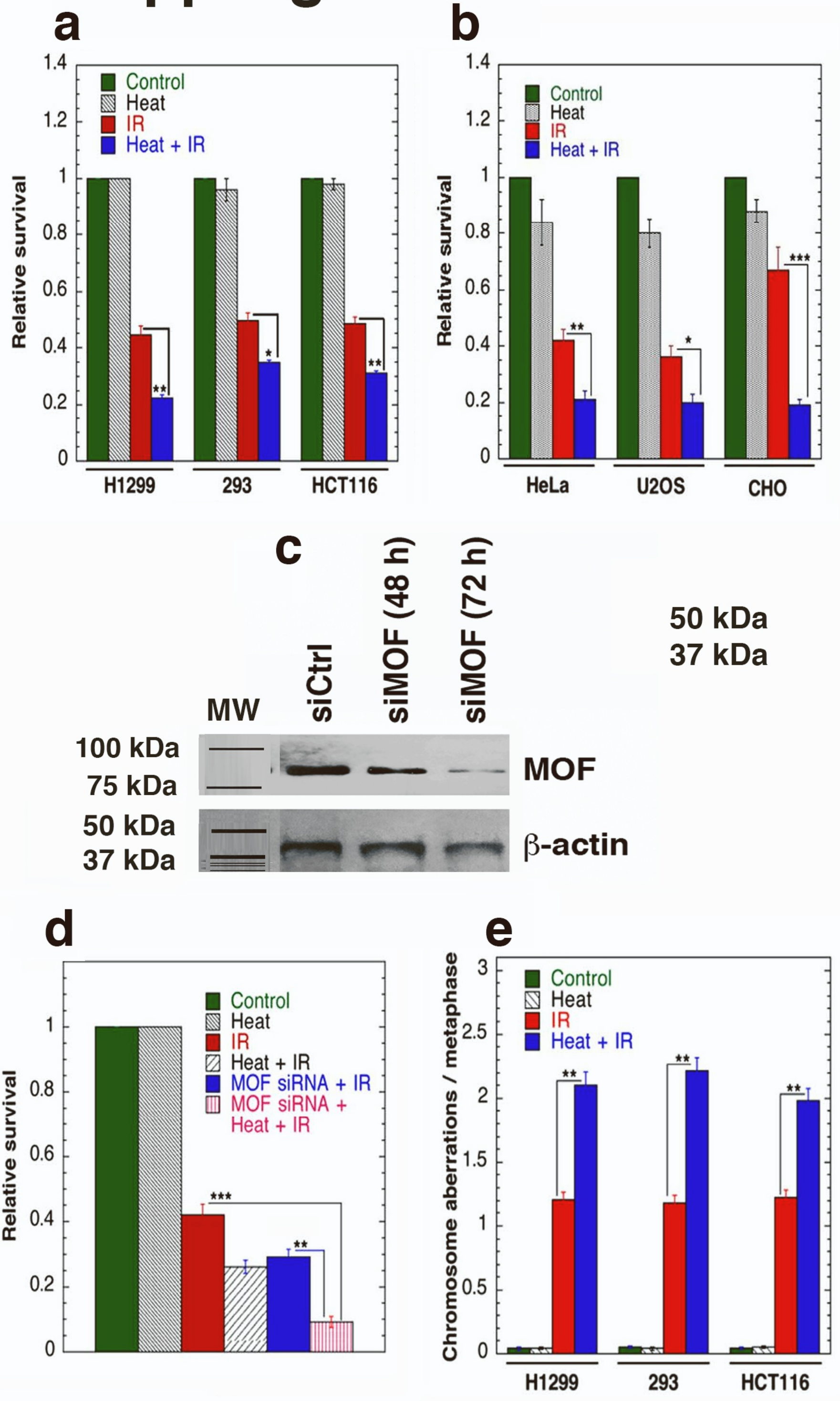

a
Control
Heat
IR
Heat + IR
Relative survival
H1299
293
HCT116
b
Control
Heat
IR
Heat + IR
Relative survival
HeLa
U2OS
CHO
c
MW
siCtrl
siMOF (48 h)
siMOF (72 h)
100 kDa
75 kDa
50 kDa
37 kDa
MOF
β-actin
50 kDa
37 kDa
d
Control
Heat
IR
Heat + IR
MOF siRNA + IR
MOF siRNA + Heat + IR
Relative survival
e
Control
Heat
IR
Heat + IR
Chromosome aberrations / metaphase
H1299
293
HCT116

Supp Fig 3

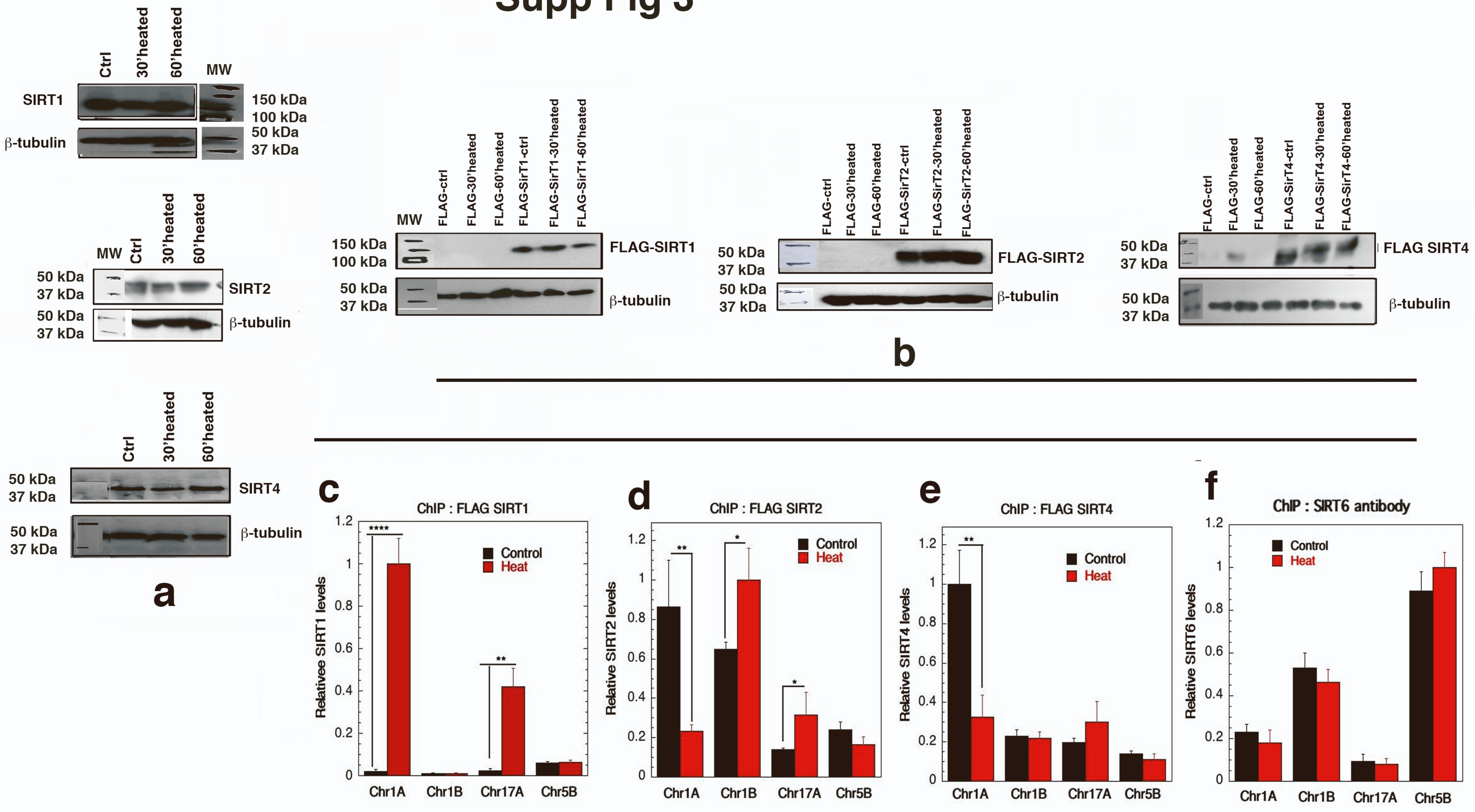

Ctrl
30'heated
60'heated
MW
SIRT1
β-tubulin
150 kDa
100 kDa
50 kDa
37 kDa
SIRT2
SIRT4
FLAG-ctrl
FLAG-30'heated
FLAG-60'heated
FLAG-SirT1-ctrl
FLAG-SirT1-30'heated
FLAG-SirT1-60'heated
FLAG-SIRT1
FLAG-SirT2-ctrl
FLAG-SirT2-30'heated
FLAG-SirT2-60'heated
FLAG-SIRT2
FLAG-SirT4-ctrl
FLAG-SirT4-30'heated
FLAG-SirT4-60'heated
FLAG SIRT4
a
b
c
ChIP : FLAG SIRT1
Relativee SIRT1 levels
d
ChIP : FLAG SIRT2
Relative SIRT2 levels
e
ChIP : FLAG SIRT4
Relative SIRT4 levels
f
ChIP : SIRT6 antibody
Relative SIRT6 levels
Control
Heat
Chr1A
Chr1B
Chr17A
Chr5B

Supp Fig. 4

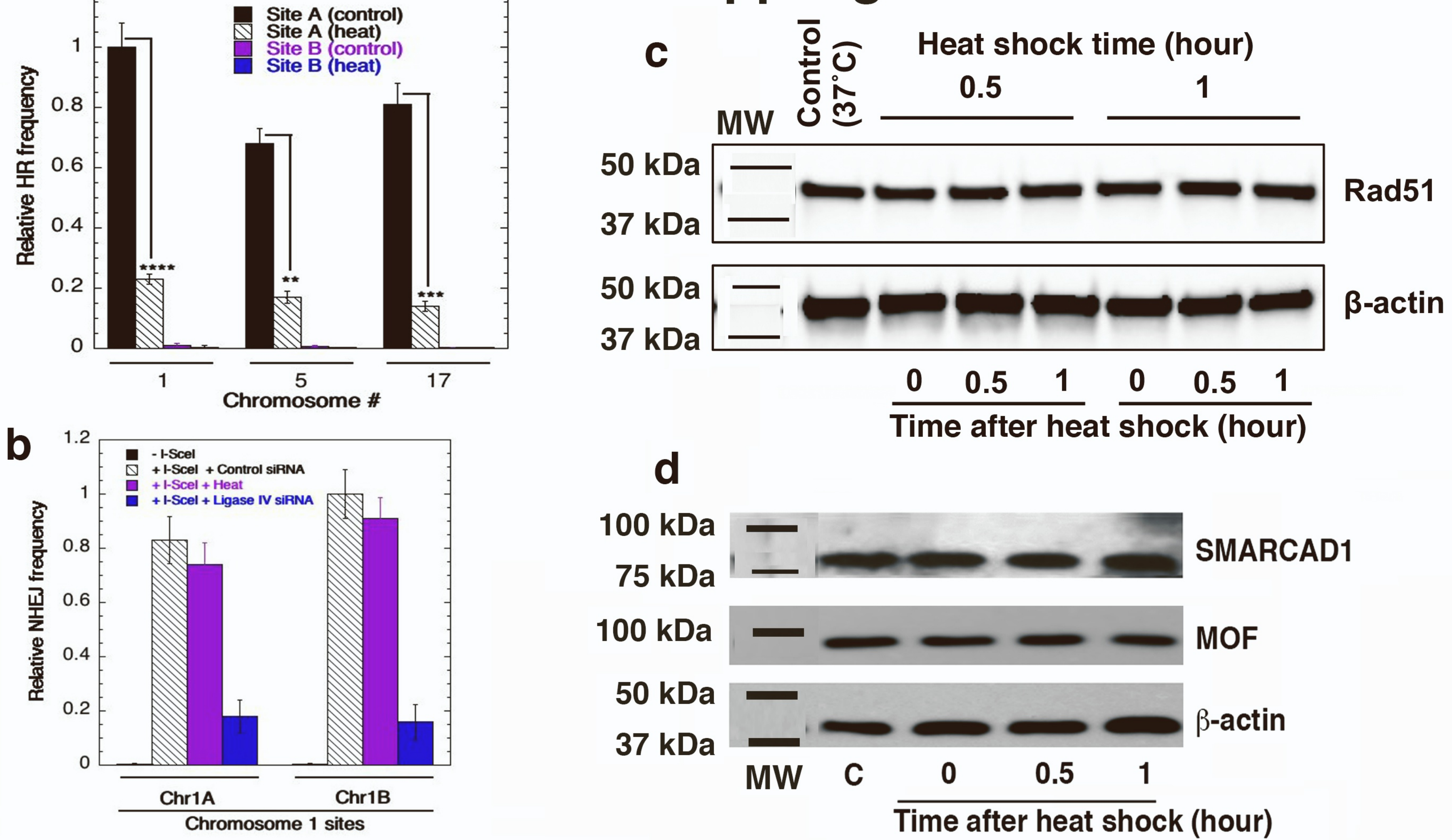
a
Relative HR frequency
1.2
1
0.8
0.6
0.4
0.2
0
Site A (control)
Site A (heat)
Site B (control)
Site B (heat)
****
**
***
1
5
17
Chromosome #
b
Relative NHEJ frequency
- I-SceI
+ I-SceI + Control siRNA
+ I-SceI + Heat
+ I-SceI + Ligase IV siRNA
Chr1A
Chr1B
Chromosome 1 sites
c
MW
Control (37°C)
Heat shock time (hour)
0.5
1
50 kDa
37 kDa
Rad51
β-actin
0
0.5
1
0
0.5
1
Time after heat shock (hour)
d
100 kDa
75 kDa
SMARCAD1
100 kDa
MOF
50 kDa
37 kDa
β-actin
MW
C
0
0.5
1
Time after heat shock (hour)

Supp Fig. 5

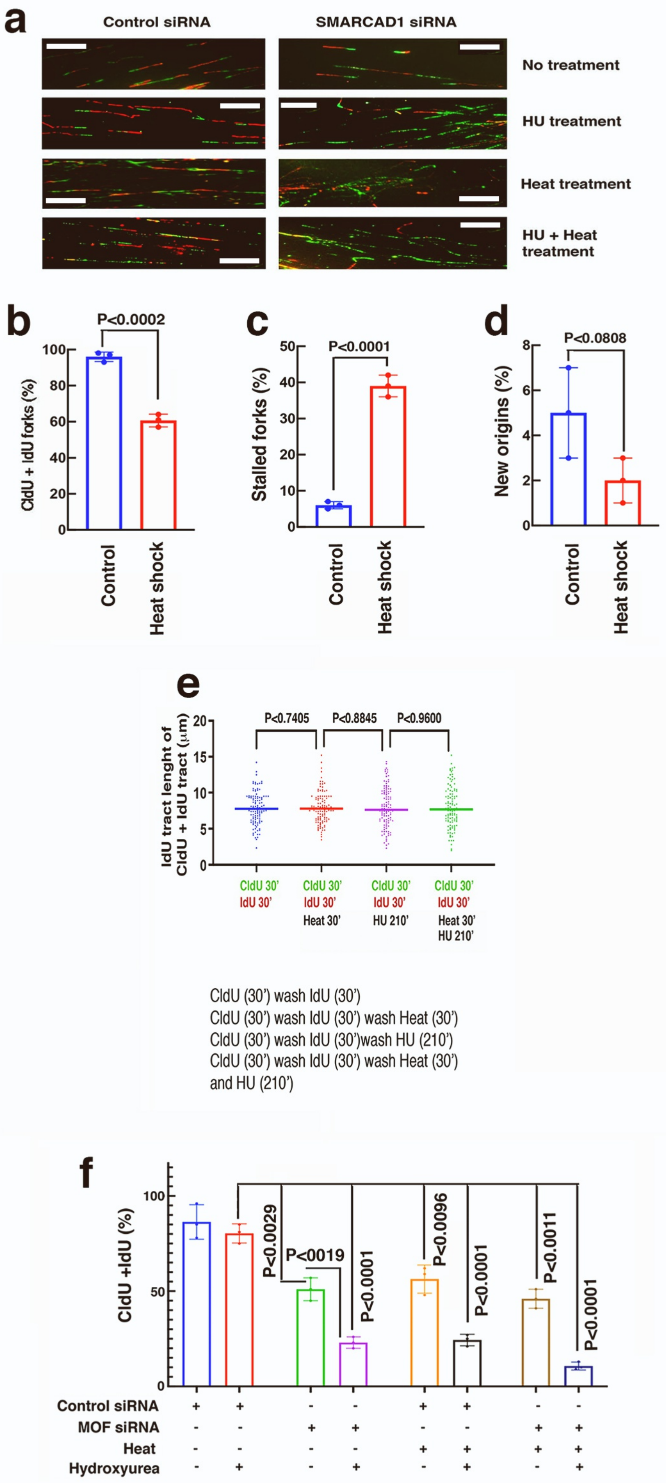
a
Control siRNA
SMARCAD1 siRNA
No treatment
HU treatment
Heat treatment
HU + Heat treatment
b
P<0.0002
CldU + IdU forks (%)
Control
Heat shock
c
P<0.0001
Stalled forks (%)
Control
Heat shock
d
P<0.0808
New origins (%)
Control
Heat shock
e
P<0.7405
P<0.8845
P<0.9600
IdU tract lenght of CldU + IdU tract (μm)
CldU 30' IdU 30'
CldU 30' IdU 30' Heat 30'
CldU 30' IdU 30' HU 210'
CldU 30' IdU 30' Heat 30' HU 210'
CldU (30') wash IdU (30')
CldU (30') wash IdU (30') wash Heat (30')
CldU (30') wash IdU (30')wash HU (210')
CldU (30') wash IdU (30') wash Heat (30') and HU (210')
f
CldU +IdU (%)
P<0.0029
P<0019
P<0.0001
P<0.0096
P<0.0001
P<0.0011
P<0.0001
Control siRNA
MOF siRNA
Heat
Hydroxyurea

Supp Fig. 6

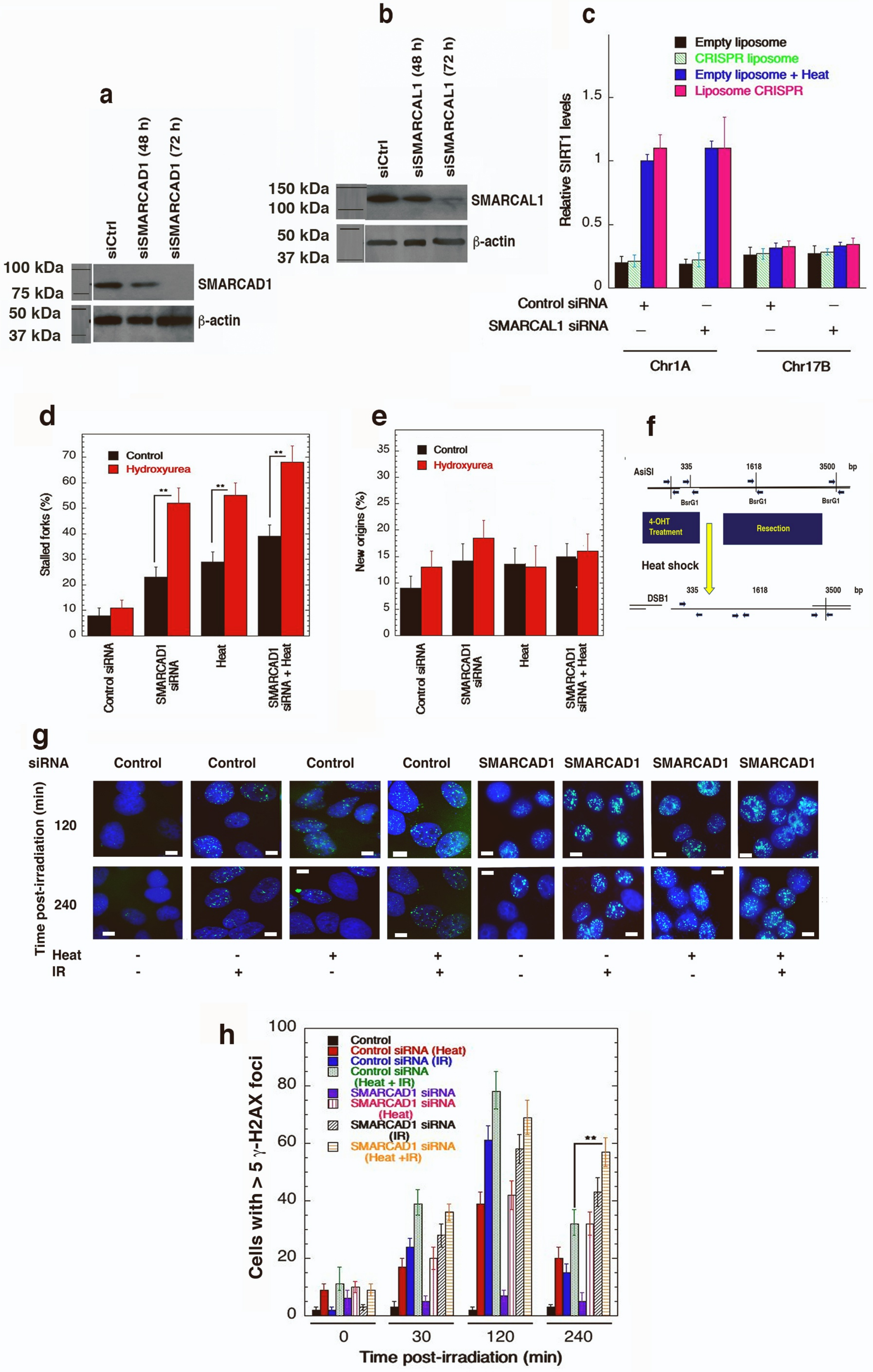
a
siCtrl
siSMARCAD1 (48 h)
siSMARCAD1 (72 h)
100 kDa
75 kDa
50 kDa
37 kDa
SMARCAD1
β-actin
b
siCtrl
siSMARCAL1 (48 h)
siSMARCAL1 (72 h)
150 kDa
100 kDa
50 kDa
37 kDa
SMARCAL1
β-actin
c
Empty liposome
CRISPR liposome
Empty liposome + Heat
Liposome CRISPR
Relative SIRT1 levels
0
0.5
1
1.5
Control siRNA
SMARCAL1 siRNA
Chr1A
Chr17B
d
Control
Hydroxyurea
Stalled forks (%)
0
10
20
30
40
50
60
70
**
Control siRNA
SMARCAD1 siRNA
Heat
SMARCAD1 siRNA + Heat
e
Control
Hydroxyurea
New origins (%)
0
5
10
15
20
25
30
35
Control siRNA
SMARCAD1 siRNA
Heat
SMARCAD1 siRNA + Heat
f
AsiSI
335
1618
3500
bp
BsrG1
4-OHT Treatment
Resection
Heat shock
DSB1
g
siRNA
Control
SMARCAD1
Time post-irradiation (min)
120
240
Heat
IR
h
Control
Control siRNA (Heat)
Control siRNA (IR)
Control siRNA (Heat + IR)
SMARCAD1 siRNA
SMARCAD1 siRNA (Heat)
SMARCAD1 siRNA (IR)
SMARCAD1 siRNA (Heat +IR)
Cells with > 5 γ-H2AX foci
0
20
40
60
80
100
**
0
30
120
240
Time post-irradiation (min)

Supp Fig. 7

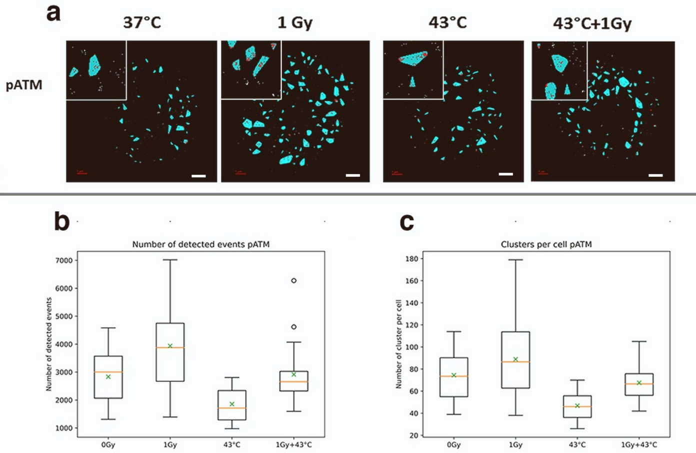
a
37°C
1 Gy
43°C
43°C+1Gy
pATM
b
Number of detected events pATM
Number of detected events
1000
2000
3000
4000
5000
6000
7000
0Gy
1Gy
43°C
1Gy+43°C
c
Clusters per cell pATM
Number of cluster per cell
20
40
60
80
100
120
140
160
180
0Gy
1Gy
43°C
1Gy+43°C
